# Noncanonical amino acid mutagenesis in response to recoding signal-enhanced quadruplet codons

**DOI:** 10.1093/nar/gkac474

**Published:** 2022-06-03

**Authors:** Yan Chen, Xinyuan He, Bin Ma, Kun Liu, Tianyu Gao, Wei Niu, Jiantao Guo

**Affiliations:** Department of Chemistry, University of Nebraska-Lincoln, Lincoln, NE 68588, USA; Department of Chemical & Biomolecular Engineering, University of Nebraska-Lincoln, Lincoln, NE 68588, USA; Department of Chemistry, University of Nebraska-Lincoln, Lincoln, NE 68588, USA; Department of Chemistry, University of Nebraska-Lincoln, Lincoln, NE 68588, USA; Department of Chemistry, University of Nebraska-Lincoln, Lincoln, NE 68588, USA; Department of Chemical & Biomolecular Engineering, University of Nebraska-Lincoln, Lincoln, NE 68588, USA; The Nebraska Center for Integrated Biomolecular Communication (NCIBC), University of Nebraska-Lincoln, Lincoln, NE 68588, USA; Department of Chemistry, University of Nebraska-Lincoln, Lincoln, NE 68588, USA; The Nebraska Center for Integrated Biomolecular Communication (NCIBC), University of Nebraska-Lincoln, Lincoln, NE 68588, USA

## Abstract

While amber suppression is the most common approach to introduce noncanonical amino acids into proteins in live cells, quadruplet codon decoding has potential to enable a greatly expanded genetic code with up to 256 new codons for protein biosynthesis. Since triplet codons are the predominant form of genetic code in nature, quadruplet codon decoding often displays limited efficiency. In this work, we exploited a new approach to significantly improve quadruplet UAGN and AGGN (N = A, U, G, C) codon decoding efficiency by using recoding signals imbedded in mRNA. With representative recoding signals, the expression level of mutant proteins containing UAGN and AGGN codons reached 48% and 98% of that of the wild-type protein, respectively. Furthermore, this strategy mitigates a common concern of reading-through endogenous stop codons with amber suppression-based system. Since synthetic recoding signals are rarely found near the endogenous UAGN and AGGN sequences, a low level of undesirable suppression is expected. Our strategy will greatly enhance the utility of noncanonical amino acid mutagenesis in live-cell studies.

## INTRODUCTION

The genetic incorporation of noncanonical amino acids (ncAAs) is a useful tool for biological studies. A general approach for the site-specific incorporation of ncAAs in live cells has been developed (1–12). This system is mainly based on suppressing one of the three nonsense triplet codons, especially the amber codon. In order to unleash the full potential of ncAA mutagenesis in biological studies and/or biomedical applications, more blank codons (codons that do not currently encode any proteinogenic amino acids) are needed. The quadruplet codon is emerging as an excellent choice. In theory, a quadruplet codon table would provide a total of 256 ‘blank’ codons that can be used to greatly expand the genetic code. However, broad applications of quadruplet codon decoding method are hampered by low decoding efficiency. Efforts have been made to improve quadruplet codon decoding efficiency by modifying the anticodon-binding pocket of aminoacyl-tRNA synthetase (13,14). We (15,16) and others (17) also reported a strategy to enhance decoding efficiency through tRNA engineering. Albeit of recent successful examples, the current system still displayed low decoding efficiency at many tested positions of a protein.

A common concern of using nonsense suppression-based ncAA mutagenesis in live cell studies is the read-through of endogenous stop codons in cells’ normal transcriptome, which could perceivably cause undesirable interference to cells’ physiology. One solution to this problem is through massive genomic recoding to replace all stop codons of interest in the host cell's genome. For example, engineered *Escherichia coli* strains (e.g. C321.ΔA.exp and B-95.ΔA) that have their amber codons replaced with ochre codons are specifically suitable for ncAA mutagenesis through the suppression of exogenously introduced amber codons (18,19). However, due to resource requirements and limitations in current genome-editing technologies, this approach is currently limited to *E. coli* and is not readily applicable to numerous mammalian cell lines that are used in biomedical research. As an alternative to the suppression of triplet nonsense codons, quadruplet codon decoding approach can partially mitigate the suppression of endogenous stop codons. For example, UAGA codon decoding would only affect 25% of regular amber codons that are followed by a nucleotide A (20).

In the present work, inspired by the fact that an efficient +1 frameshift (an apparent quadruplet codon decoding) event in nature usually requires a recoding signal that is embedded at the close proximity of the frameshift site (21), we conduct a systematic study to identify and to embed appropriate recoding signals to boost quadruplet UAGN (N = A, U, G, C) decoding efficiency. Furthermore, the possibility of suppressing endogenous amber UAG stop codons is greatly reduced due to the lack of a nearby strong recoding signal. In contrast, highly efficient decoding of exogenously introduced UAGN codons can be achieved in the presence of a proper recoding signal. In addition to UAGN codons, we also identified recoding signals for AGGN codons and gained insights in the function of recoding signals. Our approach has a general applicability to any transformable/transfectable cells, thus has broad impacts on the general field of ncAA mutagenesis.

## MATERIALS AND METHODS

### Materials and general methods

BocLys was purchased from Bachem. K-alkyne and K-alkene were synthesized by following previously reported method (22). Primers were ordered from Sigma. Restriction enzymes, Gibson Assembly reagents, restriction enzymes, and T4 DNA ligase were purchased from New England Biolabs. KOD hot start DNA polymerase was purchased from EMD Millipore. Standard molecular biology techniques (23) were used throughout. Site-directed mutagenesis was carried out using overlapping PCR. Gibson Assembly or ligation was used for cloning. *Escherichia coli* GeneHogs (Thermo Fisher Scientific Inc) were used for routine cloning and DNA propagation. *E. coli* C321.ΔA.exp (Addgene) or GeneHogs were used for libraries screening and evaluation. All solutions were prepared in deionized water further treated by Barnstead Nanopure® ultrapure water purification system (Thermo Fisher Scientific Inc.). Antibiotics were added where appropriate to the following final concentrations: ampicillin, 100 mg L^−1^; kanamycin, 50 mg L^−1^.

### Library construction

Procedures for library construction were adapted from Noren and Noren (24). To construct libraries, nine nucleotides surrounding quadruplet codons were randomized with NNN, NHN, or NNH (N = A, G, U and C; H = A, U and C; Supplementary Table S1) using PCR mutagenesis. The resulting PCR products were introduced into vectors (Supplementary Figure S1) with Gibson Assembly method (25). Primers that were used for library construction can be found in Supplementary Table S2.

### FACS screening

The screening was conducted by adapting a previously established procedure (Supplementary Figure S2) (26). In the negative screening, cells (>3 × library size) containing a desirable library were harvested from an overnight culture at 37°C, washed and resuspended in PBS, and subjected to FACS analysis (BD FACSAria II flow cytometer). Non-fluorescent cells were collected and amplified. Library DNA was purified and transformed into either C321.ΔA.exp (for Lib-UAGN-2) or GeneHogs (for Lib-UAGN-1 and Lib-AGGN). In positive screening, cells (>3 × library size) were cultivated in the presence of 5 mM BocLys at 37°C overnight, washed and resuspended in PBS, and screened by FACS. Top 1% fluorescent cells were collected. All FACS data were plotted using BD FACSDiva software and are shown in Supplementary Figure S3.

### Fluorescence analysis of bacteria culture

Cells from FACS screening were plated on LB pates containing 5 mM BocLys. Colonies that were able to produce green fluorescence were picked from the plate and used to inoculated LB media in 96-deepwell plates. After overnight cultivation at 37°C, 50 μl of culture was sub-cultured in LB media with or without 5 mM BocLys in 96-deepwell plates. After an additional 8 hr of cultivation at 37°C, 1 ml of cell culture was collected, washed, and resuspend in 1 ml of deionized water. Fluorescence and cell density measurements were conducted with a Synergy H1 Hybrid plate reader (BioTek Instrument). The fluorescence of sfGFP was monitored at *l*_ex_ = 485 nm and *l*_em_ = 510 nm. The cell density was estimated by measuring the sample absorbance at 600 nm. Values of fluorescence intensity were normalized to cell growth. The fluorescence analysis of all mutants was conducted using the same procedure with three replicates.

### Mammalian cell culture

293T cells were grown in DMEM (HyClone) media with 10% FBS (v/v) at 37°C in a humidified atmosphere of 5% CO_2_. About 10^5^ cells were seeded in a single well of a 24-well plate and cultured overnight. Transfection was conducted at 70–80% cell confluency with Lipofectamine 2000 (Life Technologies) by following the manufacturer's instructions. Six hours post transfection, ncAA of interest was added to each sample well at a final concentration of 5 mM and an equal volume of growth medium was added to each control well.

### Flow cytometry analysis and fluorescence imaging

For flow cytometry, cells were detached from plates with 0.25% trypsin, washed twice with DPBS, fixed with 4% paraformaldehyde (w/v) at room temperature for 20 min, and resuspended in DPBS. The expression of EGFP was measured using a Beckman Coulter CytoFLEX LX flow cytometer and 3 × 10^4^ cells were recorded for each sample. The flow cytometry data were analyzed using FlowJo v10 software (Tree Star). For fluorescence imaging, transfected cells were grown for an additional 48 h before being washed with DMEM base medium and DPBS, and fixed with 4% paraformaldehyde (w/v). The fixed cells were visualized by an Inverted (Olympus IX 81) confocal microscope. The samples were excited at 488 nm to acquire EGFP fluorescence images at 530/25 nm.

### Protein purification and mass spectrometry analysis

C321. ΔA.exp cells (for UAGA-1-hit and UAGN-2-hits) and GeneHogs cells (for AGGN-hits) were used for protein expression. After overnight cultivation at 37°C in LB media, 1 ml of culture was sub-cultured in 25 ml LB media with 5 mM ncAA for an additional 8 h at 37°C. Proteins were purified from cell lysates using Ni resin, separated by 15% SDS-PAGE, and stained with Coomassie blue. The protein bands of interest were excised, washed, and treated with trypsin overnight at 37°C. All mass spectrometry analyses were conducted using a Q-Exactive HF mass spectrometer (Thermo Fisher) equipped with an Acquity UPLCM-Class Peptide CSH C18 column (Waters Corp., Milford, MA). Peptide fragments were identified by searching the Mascot database.

## RESULTS

### Identification of recoding signals for UAGN with the first generation of library

**Figure 1.**
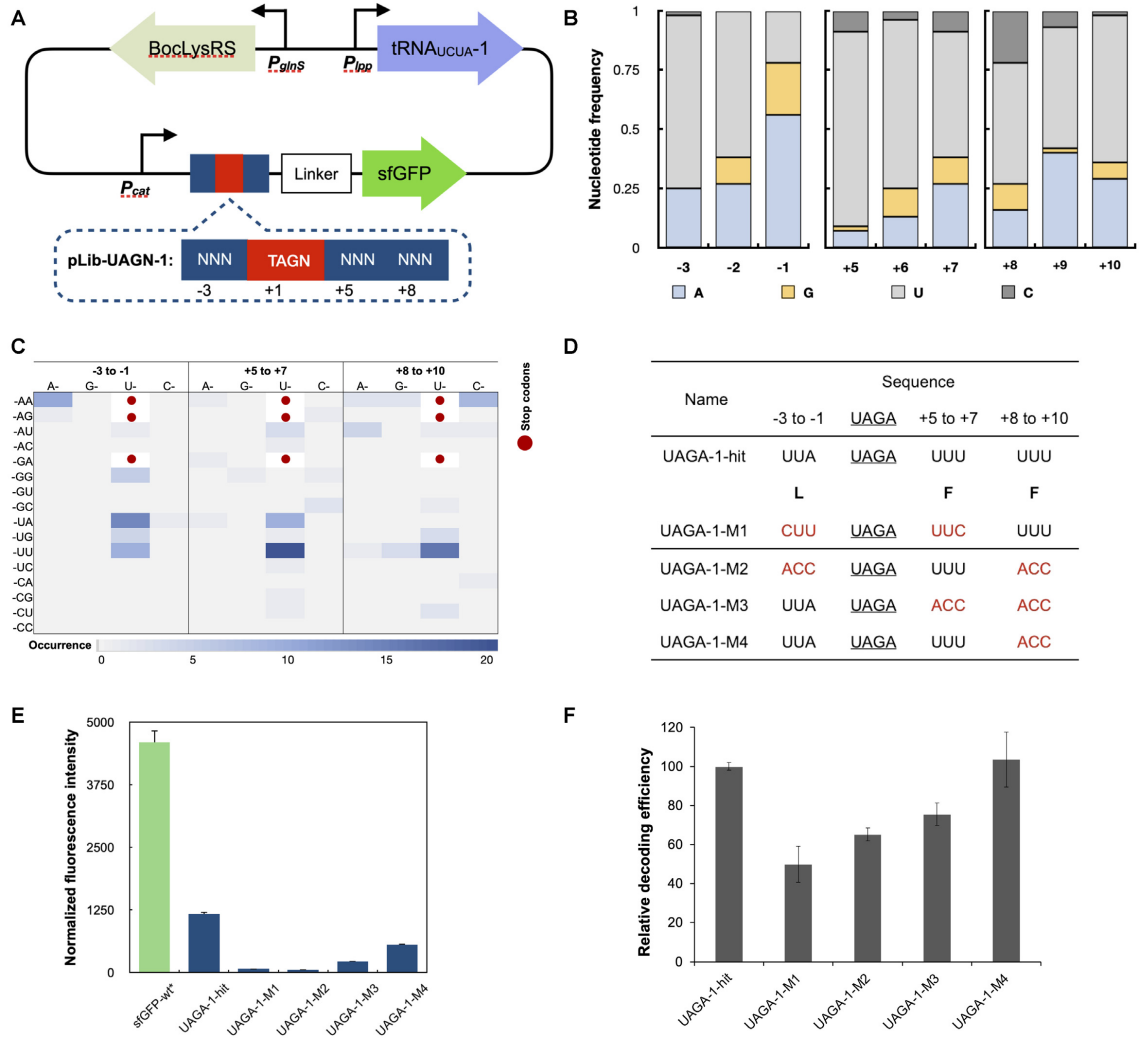
Identification of recoding signals for UAGN codons with the first generation of library. (**A**) A schematic of libraries. Randomized nucleotides are shown as N (N = A, T, G or C). (**B**) Nucleotides sequence analysis of 45 distinct hits (Supplementary Table S3) identified from the screening. (**C**) Heat map of codon preference of 45 distinct hits for UAGA decoding (Supplementary Table S3). Three stop codons are shown in red dots. Codons with higher occurrence are represented in darker blue color. (**D**) Sequences of UAGA-1-hit, and synonymous codon and other mutants. Amino acid sequences are shown in bold single-letter code. Synonymous and altered codons are labeled in red. ACC is a codon sequence that is not found in any identified recoding signals. (**E**) Fluorescence assays of UAGA-1-hit and its derivatives. Protein expressions were conducted in the presence of 5 mM BocLys. Normalized fluorescence intensity is calculated by measured fluorescence intensity/OD_600_. Raw OD_600_ values are shown in Supplementary Figure S10. (**F**) Flow cytometry analysis of UAGA-1-hit and its derivatives in 293T cells. Relative decoding efficiency was calculated by multiplying the mean fluorescence intensity by the percentage of fluorescent cells. The decoding efficiency of UAGA-1-hit was set at 100%. All FACS data were plotted using BD FACSDiva software and are shown in Supplementary Figure S5. (E and F) Data are plotted as the mean ± standard deviation (s.d.) from *n* = 3 independent experiments.

The naturally occurring recoding signals of programmed +1 frameshift are imbedded in the mRNA immediately upstream and downstream of the shift site (21). To mimic this feature, libraries were constructed by fully randomizing three nucleotides upstream and six nucleotides downstream of the UAGN codon (Figure 1A). In the first generation of design, four libraries were constructed by placing UAGN codons with randomized regions near the initiation codon and in front of a superfolder green fluorescent protein (sfGFP; Figure 1A). A flexible GGAS linker was introduced between the randomized regions and sfGFP to minimize perturbation of ncAA incorporation to protein function.

Four libraries, Lib-UAGA-1 (for decoding UAGA codon), Lib-UAGG-1 (for decoding UAGG codon), Lib-UAGU-1 (for decoding UAGU codon), and Lib-UAGC-1 (for decoding UAGC codon), were created. Each library has a theoretical diversity of 2.6 × 10^5^ and was cloned into a pLib vector that encodes a previously engineered UAGN-decoding tRNA mutant (tRNA_UCUA_) and an aminoacyl-tRNA synthetase mutant (BocLysRS) that can specifically charge tRNA_UCUA_ with Nϵ-(tert-butyloxy-carbonyl)-L-lysine (BocLys) (16). The resulting library plasmids (pLib-UAGA-1, pLib-UAGG-1, pLib-UAGC-1 and pLib-UAGU-1) were separately transformed into *E. coli* GeneHogs host with a higher than 99% coverage of the theoretical diversity of each library. Fluorescent-activated cell sorting (FACS; Supplementary Figure S2) was applied to each library in a negative screening to eliminate any fluorescent cells in the absence of BocLys. The main purpose of the negative screening is to eliminate any library members containing internal start codons or deletions that were introduced in the randomized regions and led to constitutive expressions of sfGFP. Indeed, a small fraction (<5%) of the population produced fluorescence in the absence of BocLys. A few fluorescent clones were sequenced and they were confirmed to contain either internal start codons or deletions (Supplementary Table S6). Next, non-fluorescent cells from negative selection were collected and cultivated in the presence of 5 mM BocLys before being subjected to FACS analysis in the positive screening (Supplementary Figure S2). The majority of cells were non-fluorescent, which indicated that most of the randomized sequences surrounding the UAGN codon did not lead to any detectable decoding of UAGN codons. The 1% of total sorted cells (10^4^) with the highest fluorescence intensity were collected and further analyzed on LB plates with 5 mM BocLys. All single colonies with strong fluorescence were picked for plasmid isolation and sequencing and examined for their decoding efficiency by measuring fluorescence of sfGFP expressed in the presence and absence of BocLys. A total of 45 clones were confirmed as real hits that only showed significant expression of sfGFP in the presence of BocLys. Among them, sequence convergence has been observed and 22 unique recoding signals were identified (Supplementary Table S3). Interestingly, strong recoding signals were only identified for the UAGA codon, but not for the other three UAGB (B = G, C and U) codons.

Based on the 22 unique recoding signals identified for UAGA decoding (Supplementary Table S3), a consensus sequence plot was generated. The most striking feature is the preference of A (56%) at −1 and U at all the other positions (the first nucleotide of UAGA codon was assigned as +1 position (Figure 1A and B). In addition, a strong preference of specific codons was also observed and shown in Figure 1C. UUA was the most found codon at the upstream position (−3 to −1) and UUU was the most abundant codon in the two downstream locations (+5 to +7 and +8 to +10). Indeed, the sequence, UUAUAGAUUUUUU (designated as UAGA-1-hit in Figure 1D), is the most abundant one that was found in 7 clones (Supplementary Table S3). To confirm the site-specific incorporation of BocLys in response to recoding signal-dependent UAGA codon, peptide sequencing using tandem mass spectrometry (MS/MS) was performed (Supplementary Figure S4B). Following trypsin digest of purified protein (Supplementary Figure S4A), the peptide fragment that contained the position of incorporation was analyzed. Lysine, instead of BocLys, was observed at the target position. Since the BocLysRS cannot charge its tRNA with lysine according to both literature report (27) and our previous work (16), the observed peptide that contains lysine at the target site must be derived from the cleavage of the Boc group under the mass spectrometry conditions. The carbamate cleavage of BocLys was also observed previously with electron spray ionization process in the literature (28,29). The site-specific incorporation of ncAA in response to quadruplet codons is also consistent with our observation that significantly higher protein expression was observed in the presence of ncAA than in the absence of ncAA (Figure 4B).

### Characterization and study of the identified UAGA recoding signals from the first generation library

The decoding efficiency (represented by the relative protein expression level from quadruplet codon-containing and the wild-type genes) and fidelity (represented by the fold change of normalized fluorescence intensity in the presence of BocLys over that in the absence of BocLys) of all 45 unique hits are shown in Supplementary Table S3. The positive control, a wild-type sfGFP (sfGFP-wt*, without the randomized region; Figure 1E), was expressed in the same vector as that of pLib-UAGN-1. The highest observed decoding efficiency and fidelity are 32.3% and 10.1, respectively. A representative recoding signal (in bold in Supplementary Table S3) was picked based on a balanced consideration of efficiency, fidelity, and frequency of appearance, named as UAGA-1-hit (Figure 1D), and further analyzed. Since the change of nucleotide sequence leads to amino acid changes, we first conducted a mutagenesis study to understand whether the recoding signal functions at mRNA level or at the amino acid level by replacing codons in the identified recoding signals with synonymous codons that have similar frequency of usage in *E. coli* (UAGA-1-M1 in Figure 1D). Based on fluorescence assays, changing into synonymous codons led to an over 15-fold decreases in the decoding efficiency (Figure 1E). The results suggest that the nucleotide sequence rather than the amino acid sequence plays a more important role in determining the decoding efficiency of quadruplet codons. Our mutagenesis study also indicated that nucleotides at positions −3 to −1 and +5 to +7 are the most important for UAGA decoding. In contrast, nucleotides at positions +8 to +10 play a much less significant role (comparing UAGA-1-M4 to other mutants in Figure 1D and E). Indeed, a shortened recoding signal (−3 to −1 and +5 to +7) in UAGA-1-M4 led to almost 50% decoding efficiency as that of the full-length recoding signal in the UAGA-1-hit (Figure 1E). In addition, the codon immediately upstream of UAGA has an apparently higher impact on quadruplet codon decoding than that of the codon immediately downstream of UAGA (comparing UAGA-1-M2 to UAGA-1-M3 in Figure 1E). To further validate the preference of upstream UUA and downstream UUU, two smaller libraries, Lib-UAGA-1′ (UUAUAGANNN) and Lib-UAGA-1′ (NNNUAGAUUU) were created and screened on LB agar plates in the presence of 5 mM BocLys. Again, UUAUAGAUUU was observed repeatedly with strong fluorescence intensity, further confirming the preference of this identified recoding signal (Supplementary Figure S6).

To examine if the identified recoding signals work in mammalian cells, we assessed the function of UAGA-1-hit and its derivatives in 293T cells using flow cytometry. Similar to the results derived from our studies in *E. coli* (Figure 1E), synonymous codon substitution (UAGA-M1) led to a decrease in decoding efficiency and nucleotides at positions −3 to −1 and +5 to +7 were more important than those at positions +8 to +10 (Figure 1F). However, the UAGA decoding efficiency was less impacted by the identified recoding signals.

### Identification of recoding signals for UAGN with the second generation of libraries

**Figure 2.**
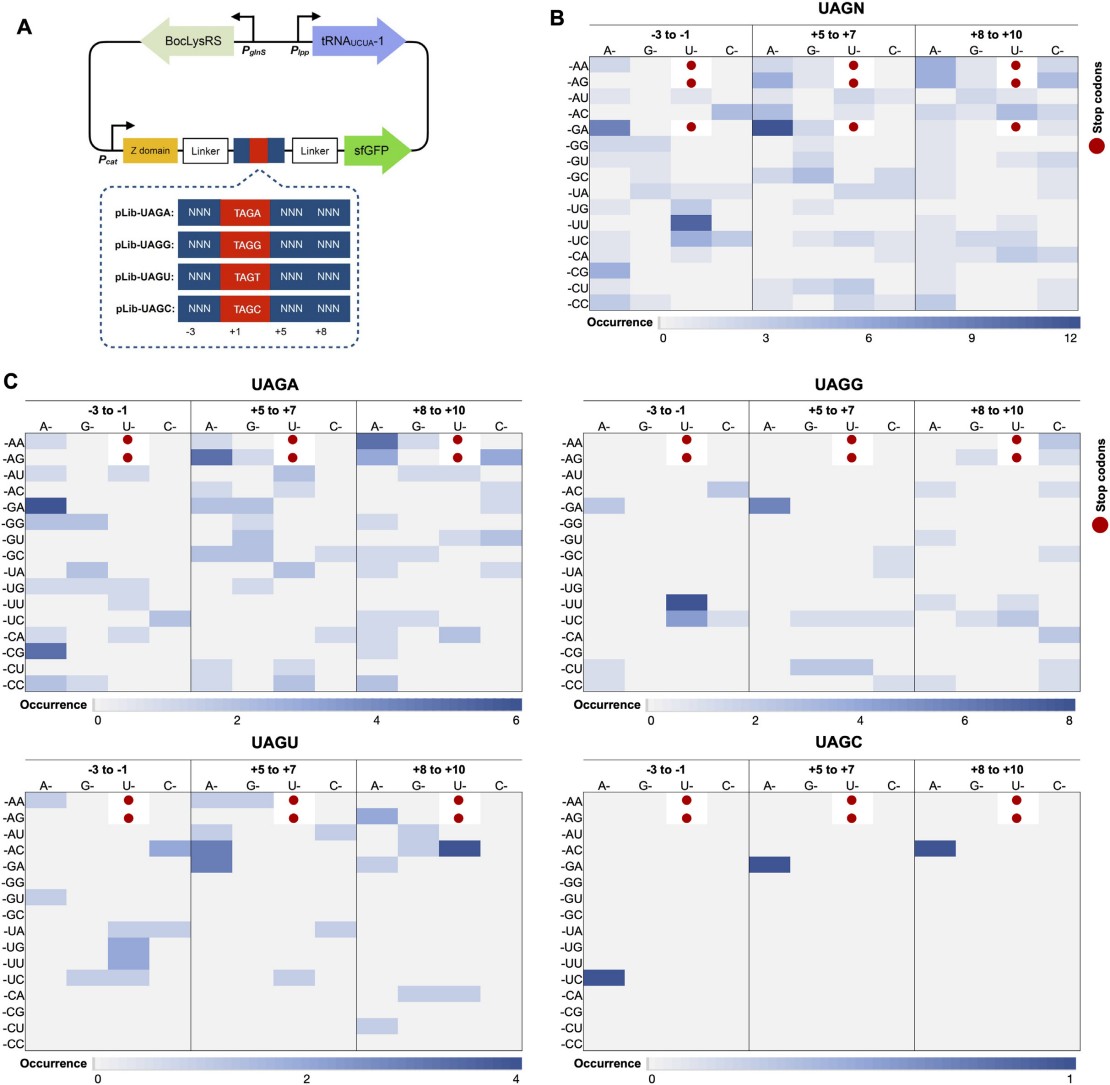
Identification of recoding signals for UAGN codons with the second generation library. (**A**) A schematic of recoding signal libraries and FACS screening. Randomized nucleotides are shown as N (N = A, T, G or C). (**B**) Heat map of codon preference of all 62 unique hits (Supplementary Table S4) identified from screening. (**C**) Heat map of codon preference of unique hits for decoding individual UAGN codons (Supplementary Table S4). (B and C) Three stop codons are shown in red dots. Codons with higher occurrence are represented in darker blue color.

Since recoding signals were only identified for the UAGA codon from the screening of the first generation libraries, we sought to design and screen new libraries. According to a previous study, efficient +1 frameshifting (an apparent quadruplet codon decoding event) only occurred when the shift site was distant from the start codon because ribosome queuing is essential to the frameshift (30). It is likely that the efficiency of UAGB (B = G, U, C) decoding near the start codon could not reach the threshold that was set for in the screening. Therefore, in the second generation of the library and reporter design, UAGN codons were sandwiched between DNA sequences that encode a Z domain and sfGFP (Figure 2A). Flexible GGAS linkers were introduced to minimize perturbation of ncAA incorporation to protein function.

Four libraries, Lib-UAGA-2 (for decoding UAGA codon), Lib-UAGG-2 (for decoding UAGG codon), Lib-UAGU-2 (for decoding UAGU codon), and Lib-UAGC-2 (for decoding UAGC codon), were constructed. Following the established FACS screening protocols (Figure 2A), no strong recoding signals was identified for UAGN codons which is possibly caused by the weak decoding efficiency of Genehog host. Library plasmids from non-fluorescent cells collected in negative sorting were amplified and transformed into C321.Δexp host (with the deletion of release factor 1 and replacement of all endogenous UAG with UAA nonsense codons) (19) and strong recoding signals were identified for all four UAGN codons. A total of 62 clones from four UAGN libraries were confirmed as real hits that only showed significant expression of sfGFP in the presence of BocLys, including 31 unique clones from Lib-UAGA-2, 18 unique clones from Lib-UAGG-2, 12 unique clones from Lib-UAGU-2, and 1 clone from Lib-UAGC-2 (Supplementary Table S4).

Our initial analysis focused on the identity of individual nucleotide in the 62 identified recoding signals. Profiles of nucleotide occurrence was built for all the recoding signals and ones for each UAGN codon separately (Supplementary Figure S7). An overall preference of the purine nucleotides (i.e. A and G) at the +5 and +6 positions was observed (Supplementary Figure S7A). Meanwhile, signals for each UAGN codon showed unique nucleotide preference (Supplementary Figure S7B). Next, we analyzed the recoding signals at the codon level. Again, heat maps of codon occurrence were generated for all the recoding signals and for each UAGN codon separately (Figure 2B, C). A few specific codons are found at high frequency in all 62 signals. Among them, the AGA codon was the most abundant one although it is a rare codon in *E. coli*. It appeared 8 times upstream (−3 to −1) and 11 times immediately downstream (+5 to +7) of UAGN codons (Figure 2B). It also appeared in the only clone that was able to efficiently decode a UAGC codon. The second most abundant codon was UUU (10 times) and all occurred at the upstream position (−3 to −1). Unlike positions −3 to −1 and +5 to +7, the codon occurrence is more balanced at the second downstream position (+8 to +10) of UAGN (Figure 2B). The analysis also showed that several codons were absent in the obtained hits, including UGG, UGC, CGG, CUG, CUU, CCG and GUU. For recoding signals of individual UAGN codons, significantly different codon preference at positions −3 to −1 and +8 to +10 was observed (Figure 2C). On the other hand, AGA was frequently occurred at the immediate downstream position (+5 to +7; Figure 2C). While AGA and ACG codons are highly abundant at the upstream position (−3 to −1) of the UAGA codon, they rarely appear at the same position for the other three quadruplet codons. At position +8 to +10, the most abundant codon for UAGA, UAGG, UAGU and UAGC are AAA, CAA/UUC/CCA, UAC and AAC, respectively.

### Characterization and study of the identified UAGA recoding signals from the second generation of library design

**Figure 3.**
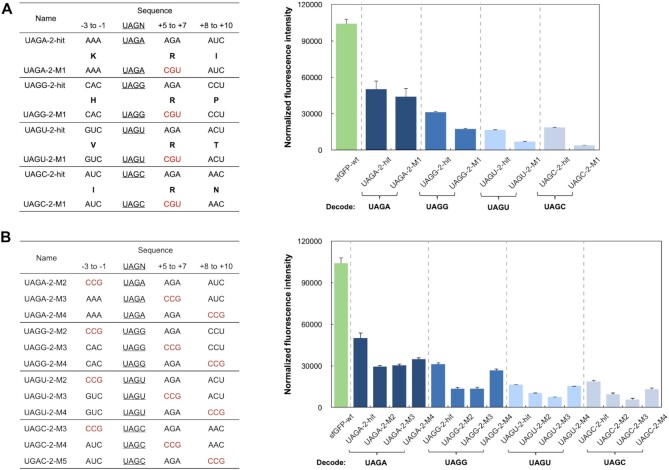
Mutational studies of recoding signals for UAGN codon decoding. (**A**) Mutational studies to assess the importance of the AGA codon at position +5 to +7. Amino acid sequences are shown in bold single letter code. Synonymous codon in each mutant is labeled in red. (**B**) Mutational studies to assess the importance of each randomized regions/codons in the identified recoding signals. Mutations are labeled in red. (A and B) The protein expression and fluorescence measurement experiments were conducted in the presence of 5 mM BocLys. Normalized fluorescence intensity is calculated by measured fluorescence intensity/OD_600_. Each data point is the average of triplicate measurements with standard deviation. Raw OD_600_ values are shown in Supplementary Figure S10B and C.

We next studied the 62 unique recoding signals for their decoding efficiency, which is represented by the relative protein expression level from quadruplet codon-containing and the wild-type genes, and the decoding fidelity, which is gauged by the fold change of normalized fluorescence intensity in the presence of BocLys over that in the absence of BocLys (Supplementary Table S4). Again, a positive control, the wild-type Z domain-sfGFP fusion protein (sf-GFP-wt, without the randomized region; Figure 3) was expressed in the same vector as that of pLib-UAGN-2. The highest observed efficiency and fidelity are 60.1% for decoding the UAGG codon and 24.9 for decoding the UAGA codon, respectively. To further understand the underlying mechanisms of the recoding signals, representative sequences for each UAGN codon were chosen based on a balanced consideration of both efficiency and fidelity, (in bold in Supplementary Table S4). These four recoding signals (fidelity, efficiency) are designated as UAGA-2-hit (8.4; 50.2%), UAGG-2-hit (12.4; 36.7%), UAGU-2-hit (15.1; 15.7%) and UAGC-2-hit (11.2; 17.5%), respectively (Supplementary Table S4). The site-specific incorporation of BocLys in response to recoding signal (UAGN-2-hit)-dependent UAGN codons was verified by mass spectrometry analysis (Supplementary Figure S4C–F).

All four selected recoding signals contained AGA at position +5 to +7, which was also the most abundant codon at this position among 62 recoding signals identified from the screening (Figure 2B, C). In the first set of mutagenesis experiments, the AGA codons were converted to CGU, a more frequently used codon for Arg (UAGN-2-M1 mutants in Figure 3A). According to protein expression experiments, except for a small decrease in UAGA-2-M1, the decoding efficiency of the other three mutants showed significantly lower fluorescence intensity than that of the original hits (Figure 3A). In our previous studies, UAGU-decoding was weak and UAGC-decoding was not detected (16).In this study we are able to observe a significant level of UAGU- and UAGC-decoding, suggesting that AGA could promoted the quadruplet codon decoding, especially the weak ones. The results further show the importance of nucleotide sequence to quadruplet decoding efficiency and suggest that AGA possibly promoted the UAGN decoding as a rare codon. To better understand the relative importance of each randomized regions/codons, three sets of mutants were constructed, including UAGN-2-M2 (−3 to −1 mutations), UAGN-2-M3 (+5 to +7 mutations), and UAGN-2-M4 (+8 to +10 mutations; Figure 3B). One original codon in each recoding signal was replaced with CCG, a codon that was not found in any identified recoding signals (Figure 2B). As shown in Figure 3B, UAGN-M4 showed the least reduced fluorescence intensity, indicating that the last codon at +8 to +10 position contributes less to the overall decoding efficiency. This observation is consistent with that of recoding signals identified from the first-generation libraries. In contrast, the fluorescence intensities of both UAGN-2-M2 and UAGN-2-M3 were significantly reduced, suggesting that codons at the −3 to −1 and +5 to +7 positions are important for efficient UAGN codon decoding (Figure 3B). Above observation indicates that an efficient recoding signal can be imbedded into mRNA with just six nucleotides surrounding a UAGN codon, which simplifies the selection of ncAA incorporation sites. Since strong recoding signals have been identified for each four UAGN codons, it would provide an expanded coverage of more amino acid sequences directly adjacent to the ncAA insertion site, which increases flexibility in selecting proper sites for ncAA mutagenesis.

Nucleotide identity analysis revealed a high frequency of the UUU codon at the upstream position (−3 to −1) of the UAGN codon (Figure 2B). To examine whether installing UUU at this position is a universal strategy to boost quadruplet codon decoding, we replaced the upstream codon in the four recoding signals with UUU (Supplementary Figure S8A). Marginal improvement in decoding efficiency was obtained (Supplementary Figure S8B). In contrast, a significant decrease in the decoding fidelity was observed for all codons except UAGA, which showed a good tolerance of an upstream UUU codon (in red, Supplementary Table S4). Further decoding experiments in the absence of BocLysRS showed substantial protein expression in cells without the UAGN-decoding tRNA (Supplementary Figure S8C). The results suggest that an upstream UUU codon likely promotes a read-through (+1 frameshift) of a quadruplet codon by the endogenous translation machinery.

### Assessment of recoding signals for UAGN decoding using different ncAAs and protein

**Figure 4.**
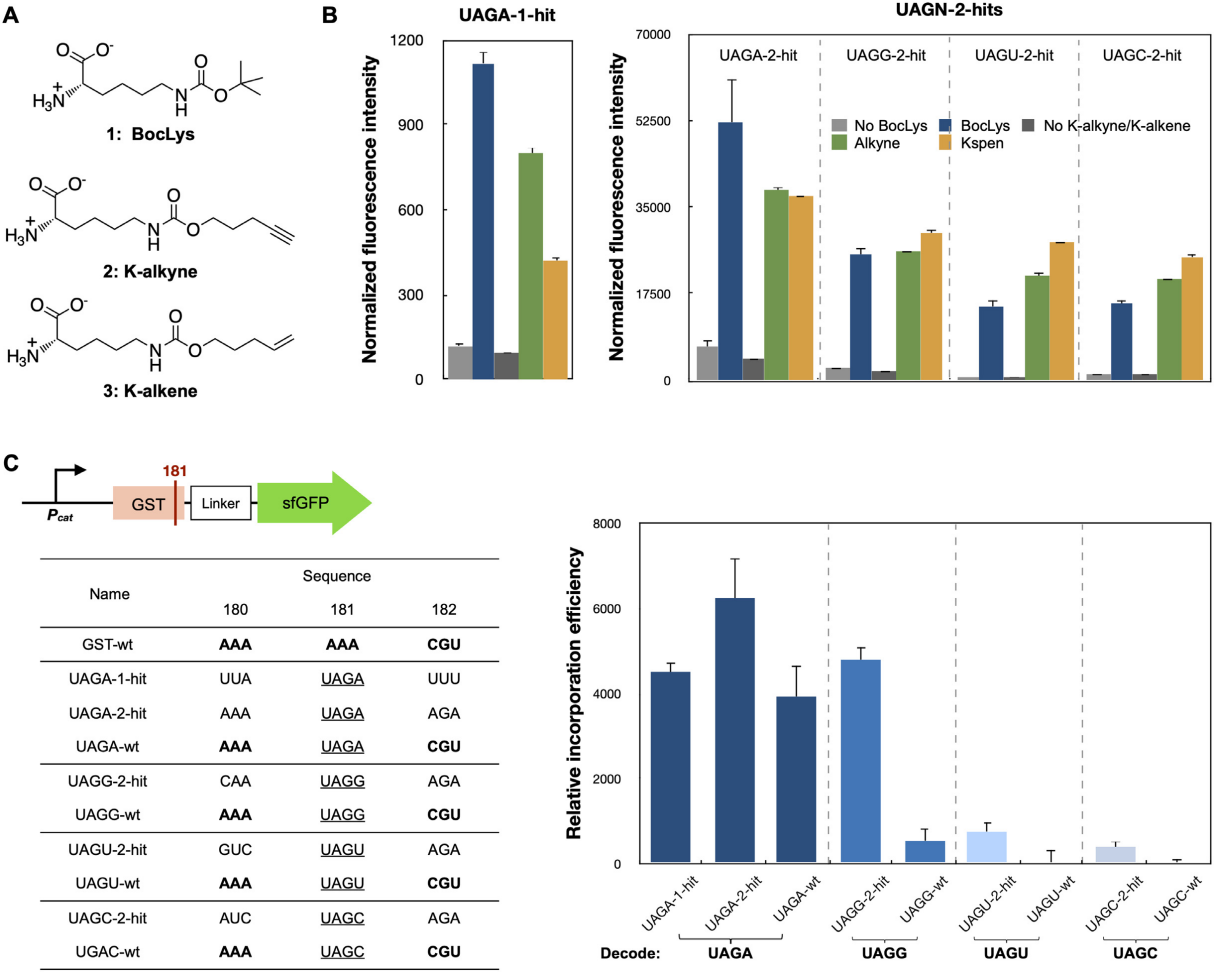
Assessment of recoding signals for UAGN decoding using different ncAAs and protein. (**A**) Chemical structures of ncAAs. 1, BocLys; 2, K-alkyne; 3, K-alkene. (**B**) Decoding recoding signal-dependent UAGN codons with BocLys, K-alkyne, and K-alkene. (**C**) Decoding recoding signal-dependent UAGN codons in GST gene. In each GST-sfGFP-UAGN mutant, position K181 of GST was mutated into UAGN codon, and positions K180 and R182 were mutated to representative recoding signals. The protein expression and fluorescence measurement experiments were conducted in the presence of 5 mM ncAAs. (B and C) Normalized fluorescence intensity is calculated by measured fluorescence intensity/OD_600_. Each data point is the average of triplicate measurements with standard deviation. Raw OD_600_ values are shown in Supplementary Figure S10.

To assess the generality of the identified recoding signals, we conducted two additional ncAA mutagenesis studies in response to UAGN codons. In the first study, the genetic incorporation of two additional ncAAs, K-alkyne and K-alkene (Figure 4A), was examined. To this end, the BocLysRS in the original pLib vector was replaced with a pyrrolysly-tRNA synthetase mutant, AcPKRS, for the site-specific incorporation of K-alkyne and K-alkene. As shown in Figure 4B, all examined recoding signals showed a good efficiency to incorporate these two ncAAs in response to UAGN codons. The results suggest that the identified recoding signals could be used to enhance the UAGN decoding efficiency with the genetic incorporation of ncAAs other than BocLys.

In the second study, we examined the function of recoding signals by the genetic incorporation of a ncAA in response to UAGN codons that were embedded inside of a gene encoding glutathione S-transferase (GST). Since our mutagenesis studies (Figure 3B) suggest that the immediate upstream and downstream codons are the most important for UAGN decoding, only three residues (K180-K181-R182) of GST were selected to install both the shortened recoding signals and UAGN codons. A sfGFP was fused to the C-terminal of GST with a GGAS linker for quantitative analysis of UAGN decoding using fluorescence assay (Figure 4C). The expression of GST-sfGFP variants was conducted in C321.ΔA.exp host, either in the presence or absence of BocLys. In comparison to UAGN-wt mutants that lack any recoding signals, an increase in decoding efficiency in the presence of recoding signals was observed for UAGA (1.6-fold), UAGG (9.5-fold), UAGU (26.6-fold) and UAGC (24.5-fold), respectively (Figure 4C). The above result is consistent with our previous finding that recoding signal can promote UAGN decoding, especially for the hard-to-decode codons, such as UAGU and UAGC (Figure 3A). As noted, UAGA-1-hit was less efficient to promote the UAGA decoding at position K181 in comparison to UAGA-2-hits. This is because UAGA-1-hit was identified from the first-generation library where the UAGA codon was placed near the start codon.

### Identification of recoding signals for AGGN codon decoding

To demonstrate the generality of recoding signal approach, we sought to identify recoding signals that can improve AGGN codon decoding. While AGG is a rare codon, it has much higher usage than a UAG codon. We want to show that the recoding signal approach can be applied to enhance decoding efficiency of exogenously added AGGN codons and do not generate significant toxicity (e.g. causing frameshift at endogenous AGG sites) to the host.

**Figure 5.**
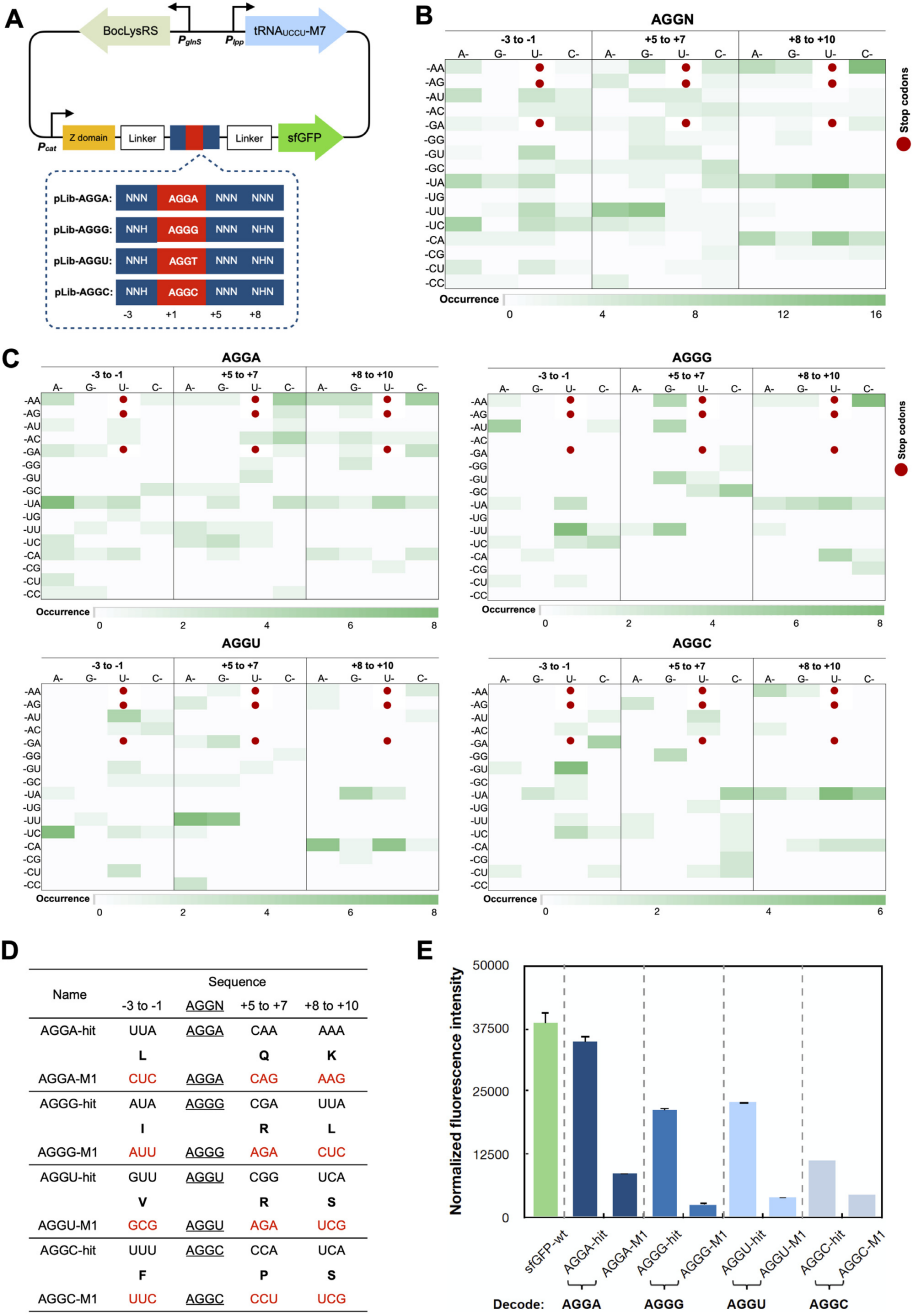
Identification of recoding signals for AGGN decoding. (**A**) A schematic of libraries. Randomized nucleotides are shown as N (N = A, T, G or C; H = A, T and C). (**B**) Heat map of codon preference of 119 unique hits for AGGN decoding (Supplementary Table S5). (**C**) Heat map of codon preference of hits for decoding individual AGGN codons (Supplementary Table S5). (B and C) Three stop codons are shown in red dots. Codons with higher occurrence are represented in darker green color. (**D**) AGGN-hits and mutants (AGGN-M1) generated from synonymous-codon replacement. Amino acid sequences are shown in bold single letter code. Codon mutations are labeled in red. (**E**) Fluorescence assays of AGGN-hits and their derivatives. The expressions were conducted in the presence of 5 mM BocLys. Normalized fluorescence intensity is calculated by measured fluorescence intensity/OD_600_. Each data point is the average of triplicate measurements with standard deviation. Raw OD_600_ values are shown in Supplementary Figure S10D.

Similar to our efforts on UAGN decoding, four libraries were constructed, including Lib-AGGA (for decoding AGGA codon), Lib-AGGG (for decoding AGGG codon), Lib-AGGU (for decoding AGGU codon), and Lib-AGGC (for decoding AGGC codon; Figure 5A). A previously engineered tRNA (tRNA_UCCU_) for the decoding of AGGN codon was used in this study (15). All four libraries were screened using *E. coli* GeneHogs as the host. While recoding signals for AGGA were readily obtained, difficulties were encountered for the AGGB (B = G, C and U) codons. Due to the palindromic feature and a high decoding efficiency of AGGA, hits obtained from Lib-AGGG, Lib-AGGU and Lib-AGGC all contained AGGA codon in the randomized region. This caused the decoding of AGG codon instead of inserted UAGB codons but still maintained the designed reading frame by decoding an AGGA codon in the randomized region. To avoid the occurrence of AGGA sequence, the randomized region of Lib-AGGG, Lib-AGGU and Lib-AGGC were modified with NNH at the upstream and NNN NHN (H = A, U or C) in the downstream regions (Figure 5A).

After FACS screening, 119 unique recoding signals (Supplementary Table S5) were identified, including 38 clones from Lib-AGGA, 28 clones from Lib-AGGG, 29 clones from Lib-AGGU, and 24 clones from Lib-AGGC. 19 clones from Lib-AGGC, and 18 clones from Lib-AGGU. The nucleotide frequency plots of recoding signals for AGGN decoding are shown in Supplementary Figure S9. The most notable feature was the strong preference of A (87%) at +10 position although this is the most distanced nucleotide from AGGN codons in the randomized region. In contrast to that of UAGN codons, the preference for specific codons was not found in the identified recoding signals surrounding AGGN codons (Figure 5B, C). In order to see if recoding signals of AGGN function similarly to those of UAGN codons, we selected one strong recoding signal identified from each library (AGGN-hits) and conducted mutagenesis studies using synonymous codons (Figure 5D). The site-specific incorporation of BocLys in response to recoding signal (AGGN-2-hit)-dependent AGGN codons was verified by mass spectrometry analysis (Supplementary Figure S4G–J). In comparison to AGGN-hits, the decoding efficiency of AGGN-M1 mutants reduced remarkably (Figure 5E). This is consistent to what we observed for UAGN decoding, confirming that the identified recoding signal function at the mRNA level. While all AGGN codons could be well decoded with recoding signals, AGGA decoding displayed the highest efficiency. With a recoding signal, the protein expression level can reach 98% of that of wild-type protein (Figure 5E). Albeit with high decoding efficiency and more frequent appearance of endogenous UAGN codons, no significant impact on cell growth was observed (Supplementary Figure S12).

## DISCUSSION

We have identified recoding signals that greatly enhanced efficiency for UAGN and AGGN codon decoding. While recoding signals for UAGN codons were identified from C321.ΔA.exp host, they should work in a similar but more robust strain, B-95.ΔA, for protein expression. We also showed that a representative recoding signal worked in mammalian cells although its impact on decoding efficiency was less significant than that in C321.ΔA.exp. This is likely due to variations in translation machinery between prokaryotes and eukaryotes. Direct screening in eukaryotes may be needed to identify recoding signals that are optimized for quadruplet codon decoding in mammalian cells. The recoding signals for AGGN decoding were screened and examined in GeneHogs cells that contain thousands of endogenous AGGN codons. We observed nearly wild-type protein expression levels.

According to our mutagenesis studies, these recoding signals function at mRNA level regardless of the position or the identity of quadruplet codons. However, we observed significant differences in sequence of recoding signals in response to the location and the identity of quadruplet codons. For UAGN codons, the frequency of codon usage in the identified recoding signals are not consistent with that of *E. coli* host, indicating a bias of nucleotides in the upstream and downstream of quadruplet codons. In contrast to that of UAGN codons, no strong codon bias was found in the identified recoding signals for AGGN codons.

When UAGN codons were placed near the initiation codon, efficient decoding was only observed for the UAGA codon. The most frequently found recoding signal was UUAUAGAUUUUUU and a synergistic influence of the immediate upstream UUA and downstream UUU codons was validated by mutagenesis studies (Figure 1D, E). The strong preference of multiple downstream Us was distinct for decoding a UAGA codon near the translation initiation site. The upstream nucleotides of the A-site codon interact with ribosomal proteins and 16s rRNA when mRNA enters through a tunnel between the head and shoulder of the ribosome subunit (31,32). These interactions could align the 3′ of mRNA towards the A site (33–35) and may play a critical role in the translation initiation (36,37). Therefore, when translation pause is absent because of the short distance between quadruplet and start codon, these Us may improve the decoding efficiency by correctly positioning the mRNA movement through their interactions with the ribosome.

When UAGN codons are located away from the initiation codon, an AGA rare codon is frequently found immediately downstream of quadruplet codons and occurred in the only clone that was able to efficiently decode a UAGC codon. The decoding efficiency decreased significantly when this AGA codon was replaced with a CGT codon, a more frequently used synonymous codon for Arg. Such decrease in decoding efficiency was also observed when AGA was replaced with a CCG, a non-synonymous but relatively more frequently used codon. These observations confirmed the essential role of this downstream rare AGA codon in the recoding process. Indeed, the translation pause has been proposed to play an important role in the +1 frameshifting (38). In one example, an insufficient recognition of a UGA codon due to a low concentration of release factor 2 (RF2) is critical to a programmed +1 frameshifting that leads to an increase in RF2 synthesis in *E. coli* (39,40). In another example, a high level of +1 frameshifting was observed at an AGA-AGA sequence in an overexpressed mRNA due to the rareness of AGA-decoding tRNAs in *E. coli* (41). Hence, a possible explanation of the frequent occurrence of this downstream AGA codon is its ability to cause a translation pause due to a slow entry of A-site tRNA (42). The crystal structure of the EF-G-ribosome complex also revealed that the flexibility of the downstream mRNA chain caused by a vacant A-site resulted in altered interactions between mRNA and ribosome (43), which may increase the change for the shift of reading frame. Ribosome queuing during translation is another consequence of the appearance of rare codons, which has been reported to affect the level of +1 frameshifting (44). Since the stalling-induced +1 frameshifting requires multiple ribosomes to load on the mRNA, this process is dependent on the location of the stalling sequence (30). This is consistent with our finding that the downstream preference of rare codons was not observed when UAGA was placed near the initiation codon, where ribosome queuing does not happen.

U is frequently found in recoding signals upstream of all examined quadruplet codons. It is likely that U has a unique interaction with the ribosome to stimulate quadruplet codon decoding. Indeed, A1493 of 16s rRNA is located close to the upstream region of the A-site codon (45) and it has been reported to involve in the codon-anticodon helix interaction (46). Furthermore, the interaction between A1503 and the upstream nucleotides of the P-site codon is also critical to maintaining the mRNA frame during the translocation (43). In contrast to that of UAGN decoding, a profound preference of nucleotides or codons was not observed in recoding signals of AGGN codons. The strong preference of A at +10 position is possibly resulted from its favorable interaction with 16s rRNA residue C1397 (43,47). The absence of downstream codon preference suggests a different frameshifting mechanism between UAGN and AGGN codons. More studies are underway to understand the role of recoding signals in UAGN and AGGN decoding.

In summary, our findings provide a new approach for designing ncAA mutagenesis in response to quadruplet codons. Recoding signals could be used to significantly boost the efficiency of quadruplet codon decoding. Indeed, with representative recoding signal UAGA-2-hit (Figure 3A) and AGGA-hit (Figure 4E), the expression level of the mutant protein reached 48% and 98% of that of the wild-type protein, respectively. It also represented a 2- to 3-fold higher expression than when amber suppression is used for the same protein at the same incorporation site, and therefore a significant reduction in the level of unwanted truncated proteins. More importantly, since strong recoding signals are rarely found near AGG and UAG codons in a host, a low-level suppression of endogenous AGGN and UAGN sequences is expected. Indeed, highly efficient decoding of exogenously introduced UAGN codons did not lead to any apparent growth defect (Supplementary Figure S11). Therefore, our approach will significantly mitigate concerns over using ncAA mutagenesis in live cell studies. While the recoding signal approach may not work for every ncAA mutagenesis event since it limits the surrounding nucleotide/amino acid sequences. As each of the UAGN and AGGN codons has different recoding signals, it provides us with an expanded pool of recoding signals that can cover more amino acid sequences directly adjacent to the ncAA insertion site. This will significantly minimize the chance of introducing mutations to the target protein due to the needs to embed recoding signals, and therefore will broaden suitable incorporation sites of ncAAs in response to quadruplet codons. Although the present work focuses on *E. coli* as host, our strategy has a general applicability to any transformable/transfectable cells and can be readily adopted by other researchers, thus has broad impacts on the general field of ncAA mutagenesis.

## DATA AVAILABILITY

All data generated or analyzed during this study are included in this article and its Supplementary Information.

## Supplementary Material

gkac474_Supplemental_File
