# Supplementary material for: Noncanonical amino acid mutagenesis in response to recoding signal-enhanced quadruplet codons
Source: Nucleic Acids Res. 2022 Jun 3;50(16):e94. doi: 10.1093/nar/gkac474 (PMC9458425; doi:10.1093/nar/gkac474)
Supplement: gkac474_Supplemental_File [file gkac474_supplemental_file.pdf]

SUPPLEMENTARY INFORMATION

Noncanonical amino acid mutagenesis in response to recoding signal-enhanced quadruplet codons

Yan Chen[1], Xinyuan He[2], Bin Ma[1], Kun Liu[1], Tianyu Gao[1], Wei Niu[2,3*], Jiantao Guo[1,3*]

[1] Department of Chemistry, University of Nebraska-Lincoln, Lincoln, Nebraska, 68588, United States.
[2] Department of Chemical & Biomolecular Engineering, University of Nebraska-Lincoln, Lincoln, Nebraska, 68588, United States.
[3] The Nebraska Center for Integrated Biomolecular Communication (NCIBC), University of Nebraska-Lincoln, Lincoln, Nebraska, 68588, United States.

* To whom correspondence should be addressed. Tel: +1 4024723525; Email: jguo4@unl.edu Correspondence may also be addressed to Wei Niu. Tel: +1 4024725348; Email: wniu2@unl.edu

Supplementary Information

Table of Contents

**Supplementary Table 1.**	Page 3
**Supplementary Table 2.**	Page 5
**Supplementary Table 3.**	Page 7
**Supplementary Table 4.**	Page 8
**Supplementary Table 5.**	Page 10
**Supplementary Table 6.**	Page 13
**Plasmid construction.**	Page 14
**Supplementary Figure 1**	Page 15
**Supplementary Figure 2.**	Page 16
**Supplementary Figure 3**	Page 17
**Supplementary Figure 4**	Page 19
**Supplementary Figure 5**	Page 21
**Supplementary Figure 6**	Page 22
**Supplementary Figure 7**	Page 23
**Supplementary Figure 8**	Page 24
**Supplementary Figure 9**	Page 25
**Supplementary Figure 10**	Page 26
**Supplementary Figure 11**	Page 27
**Supplementary Figure 12**	Page 28
**References**	Page 29

Supplementary Table 1. List of plasmids.

Plasmids	Characteristics	Vector/Source
pLib-wt' (sfGFP-wt')	Kan^R , sfGFP, $tRNA_{UCUA}$-1, BocLysRS	pBK-BocLysRS (1)
pLib-UAGA-1	Insert NNNTAGANNN NNN in the N terminal of sfGFP	pLib-wt'
pLib-UAGA-1-hit	Random region sequence: TTATAGATTTTTT	pLib-wt'
pLib-UAGA-1-M1	Random region sequence: CCTTAGATTCTTT	pLib-wt'
pLib-UAGA-1-M2	Random region sequence: ACCTAGATTTACC	pLib-wt'
pLib-UAGA-1-M3	Random region sequence: TTATAGAACCACC	pLib-wt'
pLib-UAGA-1-M4	Random region sequence: TTATAGATTTACC	pLib-wt'
pEGFP	Amp^R, sfGFP fused with N-temrinal Z domain, $tRNA_{UCUA}$-1	pEGFP-Tyr40TAGA (2)
pEGFP-UAGA-1-hit	Random region sequence: TTATAGATTTTTT	pEGFP
pEGFP-UAGA-1-M1	Random region sequence: CCTTAGATTCTTT	pEGFP
pEGFP-UAGA-1-M2	Random region sequence: ACCTAGATTTACC	pEGFP
pEGF-UAGA-1-M3	Random region sequence: TTATAGAACCACC	pEGFP
pEGFP-UAGA-1-M4	Random region sequence: TTATAGATTTACC	pEGFP
pLib-UAGA-1’	Insert TTATAGANNN between Z domain and sfGFP	pLib-wt'
pLib-UAGA-1’’	Insert NNNTAGATTT between Z domain and sfGFP	pLib-wt'
pLib-wt (sfGFP-wt)	Kan^R , sfGFP fused with N-terminal Z domain, $tRNA_{UCUA}$, BocLysRS	pBK-BocLysRS (1)
pLib-UAGA	Insert NNNTAGANNN NNN between Z domain and sfGFP	pLib-wt
pLib-UAGG	Insert NNNTAGGNNN NNN between Z domain and sfGFP	pLib-wt
pLib-UAGU	Insert NNNTAGTNNN NNN between Z domain and sfGFP	pLib-wt
pLib-UAGC	Insert NNNTAGCNNN NNN between Z domain and sfGFP	pLib-wt
pLib-UAGA-2-hit	Recoding signal: AAATAGAAGAATC	pLib-wt
pLib-UAGG-2-hit	Recoding signal: CACTAGGAGACCT	pLib-wt
pLib-UAGU-2-hit	Recoding signal: GTCTAGTAGAACT	pLib-wt
pLib-UAGC-2-hit	Recoding signal: ATCTAGCAGAAAC	pLib-wt
pLib-UAGA-2-M1	Recoding signal: AAATAGACGTATC	pLib-wt
pLib-UAGA-2-M2	Recoding signal: CCGTAGAAGAATC	pLib-wt
pLib-UAGA-2-M3	Recoding signal: AAATAGACCGATC	pLib-wt
pLib-UAGA-2-M4	Recoding signal: AAATAGAAGACCG	pLib-wt
pLib-UAGA-2-M5	Recoding signal: TTTTAGAAGAATC	pLib-wt
pLib-UAGA-2-M5Δ	Recoding signal: TTTTAGAAGAATC, $\Delta tRNA_{UCUA}$-1, ΔBocLysRS	pLib-wt
pLib-UAGG-2-M1	Recoding signal: CACTAGGCGTCCT	pLib-wt
pLib-UAGG-2-M2	Recoding signal: CCGTAGGAGACCT	pLib-wt
pLib-UAGG-2-M3	Recoding signal: CACTAGGCCGCCT	pLib-wt
pLib-UAGG-2-M4	Recoding signal: CACTAGGAGACCG	pLib-wt
pLib-UAGG-2-M5	Recoding signal: TTTTAGGAGACCT	pLib-wt
pLib-UAGG-2-M5Δ	Recoding signal: TTTTAGGAGACCT, $\Delta tRNA_{UCUA}$-1, ΔBocLysRS	pLib-wt
pLib-UAGU-2-M1	Recoding signal: GTCTAGTCGTACT	pLib-wt
pLib-UAGU-2-M2	Recoding signal: CCGTAGTAGAACT	pLib-wt
pLib-UAGU-2-M3	Recoding signal: GTCTAGTCCGACT	pLib-wt
pLib-UAGU-2-M4	Recoding signal: GTCTAGTAGACCG	pLib-wt

pLib-UAGU-2-M5	Recoding signal: TTTTAGTAGAACT	pLib-wt
pLib-UAGU-2-M5Δ	Recoding signal: TTTTAGTAGAACT, ΔtRNA$_{UCUA}$-1, ΔBocLysRS	pLib-wt
pLib-UAGC-2-M1	Recoding signal: ATCTAGCCGTAAC	pLib-wt
pLib-UAGC-2-M2	Recoding signal: CCGTAGCCGTAAC	pLib-wt
pLib-UAGC-2-M3	Recoding signal: ATCTAGCCCGAAC	pLib-wt
pLib-UAGC-2-M4	Recoding signal: ATCTAGCAGACCG	pLib-wt
pLib-UAGC-2-M5	Recoding signal: TTTTAGCAGAAAC	pLib-wt
pLib-UAGC-2-M5Δ	Recoding signal: TTTTAGCAGAACC, ΔtRNA$_{UCUA}$-1, ΔBocLysRS	pLib-wt
pBocLys-tRNA$_{UCUA}$-1	Amp^R, tRNA$_{UCUA}$-1, BocLysRS	(2)
pLib2-wt	Kan^R , sfGFP fused with N-terminal Z domain, tRNA$_{UCCU}$-M7, BocLysRS-AGGA	pLib
pLib-AGGA	Insert NNNAGGANNN NNN between Z domain and sfGFP	pLib2-wt
pLib-AGGG	Insert NNHAGGGNNN NHN between Z domain and sfGFP	pLib2-wt
pLib-AGGU	Insert NNHAGGTNNN NHN between Z domain and sfGFP	pLib2-wt
pLib-AGGC	Insert NNHAGGCNNN NHN between Z domain and sfGFP	pLib2-wt
pLib AGGA-hit	Recoding signal: TTAAGGACAAAAA	pLib2-wt
pLib AGGG-hit	Recoding signal: ATAAGGGCGATTA	pLib2-wt
pLib AGGU-hit	Recoding signal: GTTAGGTCGGTCA	pLib2-wt
pLib AGGC-hit	Recoding signal: TTTAGGCCCATCA	pLib2-wt
pLib AGGA-M1	Recoding signal: CTCAGGACAGAAG	pLib2-wt
pLib AGGG-M1	Recoding signal: ATTAGGGAGACTC	pLib2-wt
pLib AGGU-M1	Recoding signal: GCGAGGTAGATCG	pLib2-wt
pLib AGGC-M1	Recoding signal: TTCAGGCCCTTCG	pLib2-wt

Supplementary Table S2. List of primers.

Name	Sequence 5' to 3'	Usage
UAGA-1-lib-F	GAAGCTAAAATGGAGNNNTAGANNNNNNGGTGGTGCTAGCAAGGGCGAAGAG	pLib-UAGA-1
UAGA-2-lib-F	TAAGGATCTGGCGGCGCATCTNNNTAGANNNNNNGGTGGTGCTAGCAAGGGCG	pLib-UAGA-2
UAGG-2-lib-F	TAAGGATCTGGCGGCGCATCTNNNTAGGNNNNNNGGTGGTGCTAGCAAGGGCG	pLib-UAGG-2
UAGU-2-lib-F	TAAGGATCTGGCGGCGCATCTNNNTAGTNNNNNNGGTGGTGCTAGCAAGGGCG	pLib-UAGU-2
UAGC-2-lib-F	TAAGGATCTGGCGGCGCATCTNNNTAGCNNNNNNGGTGGTGCTAGCAAGGGCG	pLib-UAGC-2
AGGA-F	TAAGGATCTGGCGGCGCATCTNNNAGGANNNNNNGGTGGTGCTAGCAAGGGCG	pLib-AGGA
AGGG-lib-F	TAAGGATCTGGCGGCGCATCTNNHAGGGNNNNHNGGTGGTGCTAGCAAGGGCG	pLib-AGGG
AGGU-lib-F	TAAGGATCTGGCGGCGCATCTNNHAGGTNNNNHNGGTGGTGCTAGCAAGGGCG	pLib-AGGU
AGGC-lib-F	TAAGGATCTGGCGGCGCATCTNNHAGGCNNNNHNGGTGGTGCTAGCAAGGGCG	pLib-AGGC
UAGA’-M1-F	GAAGCTAAAATGGAGCTTTAGATTCTTTGGTGGTGCTAGC	pLib-UAGA-1-M1
UAGA’-M2-F	GAAGCTAAAATGGAGACCTAGATTTACCGGTGGTGCTAGC	pLib-UAGA-1-M2
UAGA’-M3-F	GAAGCTAAAATGGAGTTATAGAACCACCGGTGGTGCTAGC	pLib-UAGA-1-M3
UAGA’-M4-F	GAAGCTAAAATGGAGTTATAGATTTACCGGTGGTGCTAGC	pLib-UAGA-1-M4
EGFP-F-1	CCCAAGCTGGCTAGCATGGAGTTATAG	pEGFP-UAG-1-hit,-M3, M4
EGFP-F-2	CCCAAGCTGGCTAGCATGGAGCTTTAG	pEGFP-UAGA-1-M1
EGFP-F-3	CCCAAGCTGGCTAGCATGGAGACCTAG	pEGFP-UAGA-1-M2
EGFP-R	AGGCGTGTACGGTGGGAGGTCT	pEGFPand derivates
A1-F	GAAGCTAAAATGGAGTTATAGANNNGGTGGTGCTAGC	pLib-UAGA-1’
A2-F	GAAGCTAAAATGGAGNNNTAGATTTGGTGGTGCTAGC	pLib-UAGA-1’’
UAGA-M1-F	TCTGGCGGCGCATCTAAATAGACGTATCGGTGGTGCTAGC	pLib-UAGA-2-M1
UAGA-M2-F	TCTGGCGGCGCATCTCCGTAGAAGAATCGGTGGTGCTAGC	pLib-UAGA-2-M2
UAGA-M3-F	TCTGGCGGCGCATCTAAATAGACCGATCGGTGGTGCTAGC	pLib-UAGA-2-M3
UAGA-M4-F	TCTGGCGGCGCATCTAAATAGAAGACCGGGTGGTGCTAGC	pLib-UAGA-2-M4
UAGA-M5-F	TCTGGCGGCGCATCTTTTTAGAAGAATCGGTGGTGCTAGC	pLib-UAGA-2-M5
UAGG-M1-F	TCTGGCGGCGCATCTCACTAGGCGTCCTGGTGGTGCTAGC	pLib-UAGG-2-M1
UAGG-M2-F	TCTGGCGGCGCATCTCCGTAGGAGACCTGGTGGTGCTAGC	pLib-UAGG-2-M2
UAGG-M3-F	TCTGGCGGCGCATCTCACTAGGCCGCCTGGTGGTGCTAGC	pLib-UAGG-2-M3
UAGG-M4-F	TCTGGCGGCGCATCTCACTAGGAGACCGGGTGGTGCTAGC	pLib-UAGG-2-M4
UAGG-M5-F	TCTGGCGGCGCATCTTTTTAGGAGACCTGGTGGTGCTAGC	pLib-UAGG-2-M5
UAGU-M1-F	TCTGGCGGCGCATCTGTCTAGTCGTACTGGTGGTGCTAGC	pLib-UAGU-2-M1
UAGU-M2-F	TCTGGCGGCGCATCTCCGTAGTAGAACTGGTGGTGCTAGC	pLib-UAGU-2-M2
UAGU-M3-F	TCTGGCGGCGCATCTGTCTAGTCCGACTGGTGGTGCTAGC	pLib-UAGU-2-M3
UAGU-M4-F	TCTGGCGGCGCATCTGTCTAGTAGACCGGGTGGTGCTAGC	pLib-UAGU-2-M4
UAGU-M5-F	TCTGGCGGCGCATCTTTTTAGTAGAACTGGTGGTGCTAGC	pLib-UAGU-2-M5
UAGC-M1-F	TCTGGCGGCGCATCTATCTAGCCGTAACGGTGGTGCTAGC	pLib-UAGC-2-M1
UAGC-M2-F	TCTGGCGGCGCATCTCCGTAGCCGTAACGGTGGTGCTAGC	pLib-UAGC-2-M2

UAGC-M3-F	TCTGGCGGCGCATCTATCTAGCCCGAACGGTGGTGCTAGC	pLib-UAGC-2-M3
UAGC-M4-F	TCTGGCGGCGCATCTATCTAGCAGACCGGGTGGTGCTAGC	pLib-UAGC-2-M4
UAGC-M5-F	TCTGGCGGCGCATCTTTTTAGCAGAAACGGTGGTGCTAGC	pLib-UAGC-2-M5
AGGA-M1-F	TCTGGCGGCGCATCTCTCAGGACAGAAGGGTGGTGCTAGC	pLib AGGA-M1
AGGG-M1-F	TCTGGCGGCGCATCTATTAGGGAGACTCGGTGGTGCTAGC	pLib AGGG-M1
AGGU-M1-F	TCTGGCGGCGCATCTGCGAGGTAGATCGGGTGGTGCTAGC	pLib AGGU-M1
AGGC-M1-F	TCTGGCGGCGCATCTTTCAGGCCCTTCGGGTGGTGCTAGC	pLib AGGC-M1
Lib-R	AATAACTGCCCAAGCTCAGCGGTGG	pLib-wt, pLib-wt', pLib2-wt and derivatives
Lib vec-F	GCTGAGCTTGGGCAGTTATTGGTGC	pLib-wt, pLib-wt' and derivatives
Lib vec-R	AGATGCGCCGCCAGATCCCTTAGGCGCCTGAGCA	pLib-wt, pLib2-wt and derivatives
ΔZ vec-R	CTCCATTTTTAGCTTCCTTAG	pLib-wt' and derivatives

Supplementary Table 3. List of identified recoding signals from Lib-UAGA-1

Recoding signal				Occurrence	Fidelity*	Relative efficiency (%)**
**UUA**	**UAGA**	**UUU**	**UUU**	**7**	**10.1**	**25.4**
AAA	UAGA	UUA	CAA	7	9.7	26.2
UUU	UAGA	UUU	UUU	6	4.5	13.8
UGG	UAGA	UUU	GUU	3	5.7	16.1
UUG	UAGA	UAU	AAU	3	7.2	15
UGG	UAGA	UUU	GAA	2	8.8	32.3
UUA	UAGA	CGC	UUG	2	2.1	19.8
UUG	UAGA	UUG	UUU	1	2.1	19.7
UUU	UAGA	UUU	UUG	1	7.4	16.6
AAA	UAGA	UUA	CCA	1	7.9	15.4
UUU	UAGA	UCU	UUU	1	4.5	13.5
UUU	UAGA	UUU	AUU	1	5	13.4
UUA	UAGA	UUC	UUU	1	7.9	11.7
UUA	UAGA	UAC	AAA	1	5.1	11.3
AAA	UAGA	AUA	CAA	1	3.7	11
CUA	UAGA	CAG	AAU	1	4.9	10
UUA	UAGA	UUA	UGC	1	5.6	9
UUA	UAGA	AAA	CAU	1	5.4	7.7
UUA	UAGA	CGG	UAU	1	4.2	7.2
AAA	UAGA	AGA	AAA	1	3.1	5.6
UAU	UAGA	GGG	UCU	1	4.8	5.6
AAG	UAGA	UCG	UCU	1	2.8	3

* Fidelity: the fold change of normalized fluorescence intensity in the presence of BocLys over normalized fluorescence intensity in the absence of BocLys. Fluorescence intensity is normalized to cell growth.
** Relative efficiency: relative expression level of mutant to wild-type protein. The protein expression level is based on normalized fluorescence intensity.

Supplementary Table 4. List of identified recoding signals from Lib-UAGN-2

Recoding signal				Occurrence	Fidelity*	Relative efficiency (%)**
**AAA**	**UAGA**	**AGA**	**AUC**	**1**	**8.4**	**50.2**
ACG	UAGA	CCA	AGG	1	13.1	31.2
AAU	UAGA	AGA	CAG	1	5.5	29.7
AGA	UAGA	AAG	AAA	3	17.6	29.1
AUG	UAGA	ACU	AAG	1	13.8	28.7
ACC	UAGA	UCU	ACC	1	7.4	28.3
GUA	UAGA	UCC	UCA	1	21.1	26.7
AGG	UAGA	AAG	AAA	1	4.9	26.4
AGA	UAGA	UUA	ACC	1	17.0	25.9
**UUU**	**UAGA**	**UAU**	**CGA**	**1**	**24.9**	**22.3**
GGG	UAGA	GGG	CAG	1	14.5	20.0
GCC	UAGA	GAG	UGU	1	9.3	19.7
AGG	UAGA	AAA	AUA	1	4.9	19.5
ACG	UAGA	GGA	AAG	1	12.3	18.5
ACG	UAGA	GGA	AAG	1	14.4	18.3
CUC	UAGA	GGU	CGU	1	21.2	17.6
GUA	UAGA	UCC	UCA	1	18.5	17.2
ACA	UAGA	GGC	CUA	1	24.9	16.6
UAU	UAGA	AGC	GAU	1	8.3	16.4
AGA	UAGA	UAU	AAA	1	16.3	15.7
GUG	UAGA	UAC	CAC	1	24.5	13.9
UCA	UAGA	AAG	GAA	1	20.8	13.9
AGA	UAGA	AAC	ACG	1	11.2	13.1
CUC	UAGA	GGU	CGU	1	13.3	12.5
GGG	UAGA	UUA	UAU	1	5.8	12.0
ACC	UAGA	CGC	CAG	1	11.0	11.6
UUG	UAGA	GUG	GUC	1	15.1	10.8
ACG	UAGA	AGC	AGC	1	14.5	9.7
ACG	UAGA	ACC	ACA	1	12.8	9.7
UUU	UAGG	GCU	CAA	1	3.4	60.1
UUU	UAGG	GCU	CAA	1	4.2	58.4
UUU	UAGG	UCU	CAG	1	5.6	44.1
UUU	UAGG	AGA	CCA	1	4.1	42.5
ACC	UAGG	AGA	ACC	1	6.7	37.6
**CAC**	**UAGG**	**AGA**	**CCU**	**1**	**12.4**	**36.7**
CUC	UAGG	GGC	CCA	1	5.5	34.5
UUC	UAGG	GGC	GAG	1	10.4	33.6

UUU	UAGG	CUA	UUC	1	3.1	33.5
UUC	UAGG	UUC	UUC	1	9.0	31.9
ACU	UAGG	AGA	AUU	1	7.1	31.3
CAC	UAGG	GUC	CCC	1	4.6	30.7
UUC	UAGG	GGC	UUU	1	8.6	30.7
AGA	UAGG	AGA	CAC	1	9.9	30.5
UUU	UAGG	UCU	AGU	1	4.1	28.8
AGA	UAGG	CCC	CGC	1	5.4	28.6
UUC	UAGG	CUC	AAC	1	7.9	26.4
UUU	UAGG	CGC	GUC	1	3.9	25.1
UUU	UAGU	AGA	AGA	1	4.5	49.8
AAA	UAGU	AGA	AAG	1	5.0	35.6
UUU	UAGU	UUC	UAC	1	3.6	34.7
**GUC**	**UAGU**	**AGA**	**ACU**	**1**	**15.1**	**15.7**
CAC	UAGU	AAC	UAC	1	5.3	15.5
UUC	UAGU	AAU	GAU	1	7.4	13.8
UUA	UAGU	CUA	UCA	1	7.0	13.5
CUA	UAGU	AAC	AAG	1	8.0	9.1
UUG	UAGU	CAU	GAC	1	17.1	8.4
CAC	UAGU	AAC	UAC	1	5.3	7.0
UUG	UAGU	GAA	GCA	1	12.8	6.9
AGU	UAGU	AAA	UAC	1	6.4	6.4
**AUC**	**UAGC**	**AGA**	**AAC**	**1**	**11.2**	**17.5**

* Fidelity: the fold change of normalized fluorescence intensity in the presence of BocLys over normalized fluorescence intensity in the absence of BocLys. Fluorescence intensity is normalized to cell growth.
** Relative efficiency: relative expression level of mutant to wild-type protein. The protein expression level is based on normalized fluorescence intensity.

Supplementary Table 5. List of identified recoding signals from Lib-AGGN

Recoding signal				Occurrence	Fidelity*	Relative efficiency (%)**
**UUA**	**<u>AGGA</u>**	**CAA**	**AAA**	**1**	**58.1**	**90.3**
GCC	<u>AGGA</u>	CGA	CGA	1	43.1	80.3
AUA	<u>AGGA</u>	CCC	CAA	1	46.6	75.9
ACU	<u>AGGA</u>	UUC	AAA	1	34.5	73.4
AAA	<u>AGGA</u>	UAC	GAC	1	51.1	70.9
GUU	<u>AGGA</u>	AAA	GAA	1	56.1	70.7
GGA	<u>AGGA</u>	GCA	GGG	1	40.8	70.6
AUA	<u>AGGA</u>	GUC	GGA	1	39.8	70.6
AUA	<u>AGGA</u>	UAC	GGG	1	38.6	67.8
UAC	<u>AGGA</u>	CAC	ACA	1	37.7	66.2
ACA	<u>AGGA</u>	GAA	UAC	1	36.2	65.3
UCA	<u>AGGA</u>	CAG	GAG	1	40.7	63.2
CAA	<u>AGGA</u>	CAC	AUA	1	63.2	63.0
CUU	<u>AGGA</u>	AUC	UUA	1	31.0	61.5
AAA	<u>AGGA</u>	CAC	CAA	1	29.3	61.5
ACU	<u>AGGA</u>	UGG	AAA	1	35.3	61.0
UCA	<u>AGGA</u>	AUU	CCA	1	33.8	59.6
AUA	<u>AGGA</u>	AGC	GCA	1	38.4	58.8
AGA	<u>AGGA</u>	CAA	UUA	1	35.1	58.7
GUA	<u>AGGA</u>	UGU	CGA	1	44.0	56.0
AUA	<u>AGGA</u>	CAA	CAA	1	37.8	55.8
UUA	<u>AGGA</u>	CAU	UUA	1	63.7	55.3
CGC	<u>AGGA</u>	GUC	UCG	1	44.4	54.8
AAA	<u>AGGA</u>	GUA	ACA	1	40.8	54.4
ACC	<u>AGGA</u>	CAC	CGA	1	52.9	49.5
AUC	<u>AGGA</u>	AUC	AAC	1	28.1	48.5
UUA	<u>AGGA</u>	UGU	CAC	1	37.2	48.3
GCA	<u>AGGA</u>	CUA	GAA	1	45.3	46.4
CGC	<u>AGGA</u>	GUA	AUA	1	61.1	45.7
AUC	<u>AGGA</u>	GUC	CAA	1	41.7	45.4
GUA	<u>AGGA</u>	CAA	CUA	1	49.9	44.5
UAU	<u>AGGA</u>	CAG	GAC	1	31.9	41.5
AUA	<u>AGGA</u>	CUA	CCA	1	59.1	41.3
AAU	<u>AGGA</u>	GUU	CUA	1	43.1	39.1
AUA	<u>AGGA</u>	CUA	GAA	1	40.8	36.8
AGA	<u>AGGA</u>	AUU	CAA	1	49.6	35.7
UUG	<u>AGGA</u>	UUU	UUA	1	52.4	35.3

ACA	AGGA	CAA	GUA	1	40.8	35.1
**CAU**	**AGGG**	**GGU**	**CCA**	**1**	**30.7**	**45.6**
UUU	AGGG	GGU	UCA	1	34.6	41.6
UUA	AGGG	UGU	UCA	2	23.8	38.6
UUU	AGGG	GUU	GUA	1	15.2	35.8
GCA	AGGG	GGU	UCA	1	29.2	33.3
CUU	AGGG	GAA	GAA	1	22.6	32.3
UUA	AGGG	GUU	UCA	1	16.9	31.9
UUC	AGGG	GUU	UUA	1	17.3	31.6
UUU	AGGG	GAU	AUA	2	23.4	31.0
UUA	AGGG	GAA	GUA	1	20.1	30.7
AAA	AGGG	GAA	CAA	1	27.7	29.9
AUA	AGGG	CGA	UUA	5	27.4	29.7
CUC	AGGG	GUU	UUA	1	27.6	28.6
CUC	AGGG	GUU	UUA	1	23.7	27.5
UCU	AGGG	GAU	CAA	1	20.1	27.4
AAU	AGGG	CGC	CAA	1	44.0	27.3
AAU	AGGG	CGC	CAA	1	17.5	27.0
AAU	AGGG	CGC	CAA	1	29.5	26.2
AAU	AGGG	CGC	CAA	1	31.2	21.3
AAU	AGGG	CGC	CAA	1	27.1	17.7
AAU	AGGG	CGC	CAA	1	32.1	14.8
UUU	AGGG	CGG	AUU	1	14.9	13.7
**AUC**	**AGGU**	**AUU**	**ACA**	**1**	**42.0**	**65.0**
AUC	AGGU	AUU	ACA	1	22.9	49.2
AUC	AGGU	AUU	ACA	1	21.7	46.1
AUC	AGGU	AUU	ACA	1	23.1	41.0
AUC	AGGU	AUU	ACA	1	19.4	40.2
AUC	AGGU	AUU	ACA	1	19.5	34.1
AUC	AGGU	AUU	ACA	7	26.6	31.3
UAU	AGGU	GGA	GUA	1	17.0	31.2
UAU	AGGU	GGA	GUA	1	21.7	29.9
UAU	AGGU	GGA	GUA	3	23.9	29.7
UUC	AGGU	AGC	CCA	1	23.2	29.0
UCU	AGGU	GUU	UCA	1	18.5	27.8
UCU	AGGU	GUU	UCA	3	19.8	27.3
UCU	AGGU	GUU	UCA	1	20.9	26.1
CAC	AGGU	GAA	AAG	1	22.2	25.6
CAU	AGGU	AUU	UCA	1	17.3	24.8
CGC	AGGU	GUU	UCA	1	19.3	24.2

UGU	AGGU	UGU	GCA	2	21.5	24.0
**UGU**	**AGGC**	**UCU**	**GUA**	**1**	**17.1**	**31.9**
CAU	AGGC	AUU	UUA	1	16.4	21.4
UUC	AGGC	AAG	UCA	1	12.7	20.8
UGU	AGGC	GGG	AAA	1	12.5	20.3
UGU	AGGC	GGG	AAA	1	9.0	19.0
AGU	AGGC	UAU	UUA	2	30.0	18.4
CGA	AGGC	UAU	UUA	1	8.3	17.4
UGC	AGGC	GGC	CUA	1	14.4	17.1
UUU	AGGC	CCA	UCA	1	17.0	17.1
UUC	AGGC	CUA	CCA	1	9.8	17.0
GUA	AGGC	CCG	CUA	1	22.4	15.3
GUA	AGGC	CCG	CUA	3	12.5	14.7
CGA	AGGC	CUA	UUA	1	13.0	13.6
CGA	AGGC	UAU	UUA	1	10.3	13.3
CGA	AGGC	UAU	UUA	1	12.9	13.3
CUC	AGGC	CUC	AUA	1	12.2	13.1
UUA	AGGC	AUC	GCA	1	17.5	12.9
UGU	AGGC	UUG	GAA	3	13.8	12.7
UAC	AGGC	CUU	AUA	1	11.9	12.1

* Fidelity: the fold change of normalized fluorescence intensity in the presence of BocLys over normalized fluorescence intensity in the absence of BocLys. Fluorescence intensity is normalized to cell growth.
** Relative efficiency: relative expression level of mutant to wild-type protein. The protein expression level is based on normalized fluorescence intensity.

Supplementary Table 6. List of identified internal start codons and deletion mutation

Name	Sequence			
UAGA-1-P	GUG	UAGA	UUG	-UG*
UAGA-2-P	GCU	UAGA	GUG**	AGG
UAGG-2-P	UC-	UAGG	AUA	CCC
UAGU-2-P	CC-	UAGU	UUC	AAA
UAGC-2-P	GA-	UAGC	GGA	UGC

*Deletion mutations were introduced during library construction with primers containing randomized nucleotides. Deletion mutations change the reading frame and lead to the consistent expression of sfGFP.

**Prokaryotes use GUG as alternative start codon (3). The internal start codon leads to the consistent expression of sfGFP.

Plasmid construction

pLib-UAGN-1 variants. pLib-UAGN' was constructed by inserting genes encoding sfGFP and $tRNA_{UCUA}$ into pBK-BocLysRS vector (1). The expression of sfGFP and $tRNA_{UCUA}$ is controlled by P_{cat} and P_{lpp}, respectively. The P_{cat} and sfGFP fragments were amplified from pLei-sfGFP (4). The P_{lpp} and $tRNA_{UCUA}$ fragments were amplified from pBK-$tRNA_{UCUA}$ (1). Nine nucleotides surrounding quadruplet codons were randomized with NNN (N=A, G, U or C; Supplementary Table 1) using mutagenic PCR. The library was fused to N-terminal of sfGFP with a GGAS linker (GGCGGCGCATCT). The fragments of insert and the PCR-amplified pBK-BocLysRS vector were assembled by Gibson Assembly.

pLib-UAGN-2 variants. pLib-UAGN was constructed by inserting genes encoding Z-domain-sfGFP fusion protein and $tRNA_{UCUA}$ into pBK-BocLysRS vector (1). The expression of Z-domain-sfGFP and $tRNA_{UCUA}$ is controlled by P_{cat} and P_{lpp}, respectively. The P_{cat} and sfGFP fragments were amplified from pLei-sfGFP (4). The P_{lpp} and $tRNA_{UCUA}$ fragments were amplified from pBK-$tRNA_{UCUA}$ (1). The Z domain fragment was fused to the N terminal of sfGFP with a GGAS linker (GGCGGCGCATCT). The fragments of insert and the PCR-amplified pBK-BocLysRS vector were assembled by Gibson Assembly. Nine nucleotides surrounding quadruplet codons were randomized with NNN or NNH (N=A, G, U or C, H=A, U or C, Supplementary Table 1) using PCR mutagenesis.

pLib-AGGN variants. pLib-AGGN was constructed by replacing the $tRNA_{UCUA}$ and BocLysRS with $tRNA_{UCCU}$ and BocLysRS-AGGA in pLib-UAGN. The $tRNA_{UCCU}$-M7 and BocLysRS-AGGA fragments were amplified from pBK-$tRNA_{UCCU}$-M7 and pRep-BocLysRS-AGGA, respectively (5). Nine nucleotides surrounding quadruplet codons were randomized with NNN or NNH (N=A, G, U or C, H=A, U or C, Supplementary Table 1) using mutagenic PCR. The library was fused to N-terminal of sfGFP with a GGAS linker (GGCGGCGCATCT).

pEGFP-UAGA-1 variants. pEGFP derivatives were constructed by replacing EGFP 40TAGA with EGFP fused with recoding signals linked by GGAS linker using pEGFP-Tyr40TAGA (2) as the template. The gene cassette was prepared with overlap PCR and assembled with vector by HindIII and ApaI.

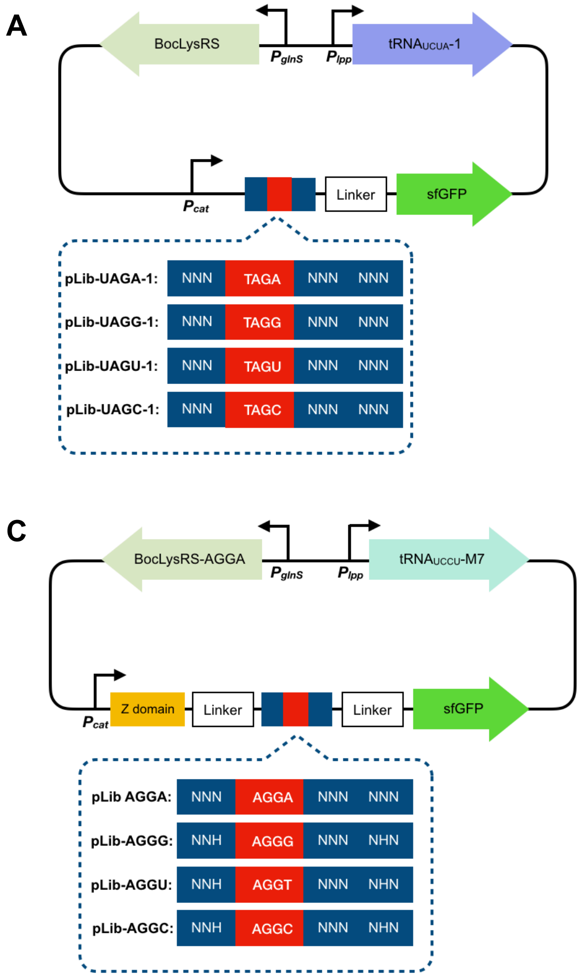

Supplementary Figure 1. (A) Plasmid design of pLib-UAGN-1 with randomized regions surrounding UAGN. **(B)** Plasmid design of pLib-UAGN-2 with randomized regions surrounding UAGN in the presence of N-terminal Z domain. **(C)** Plasmid design of pLib-AGGN with randomized regions surrounding AGGN.

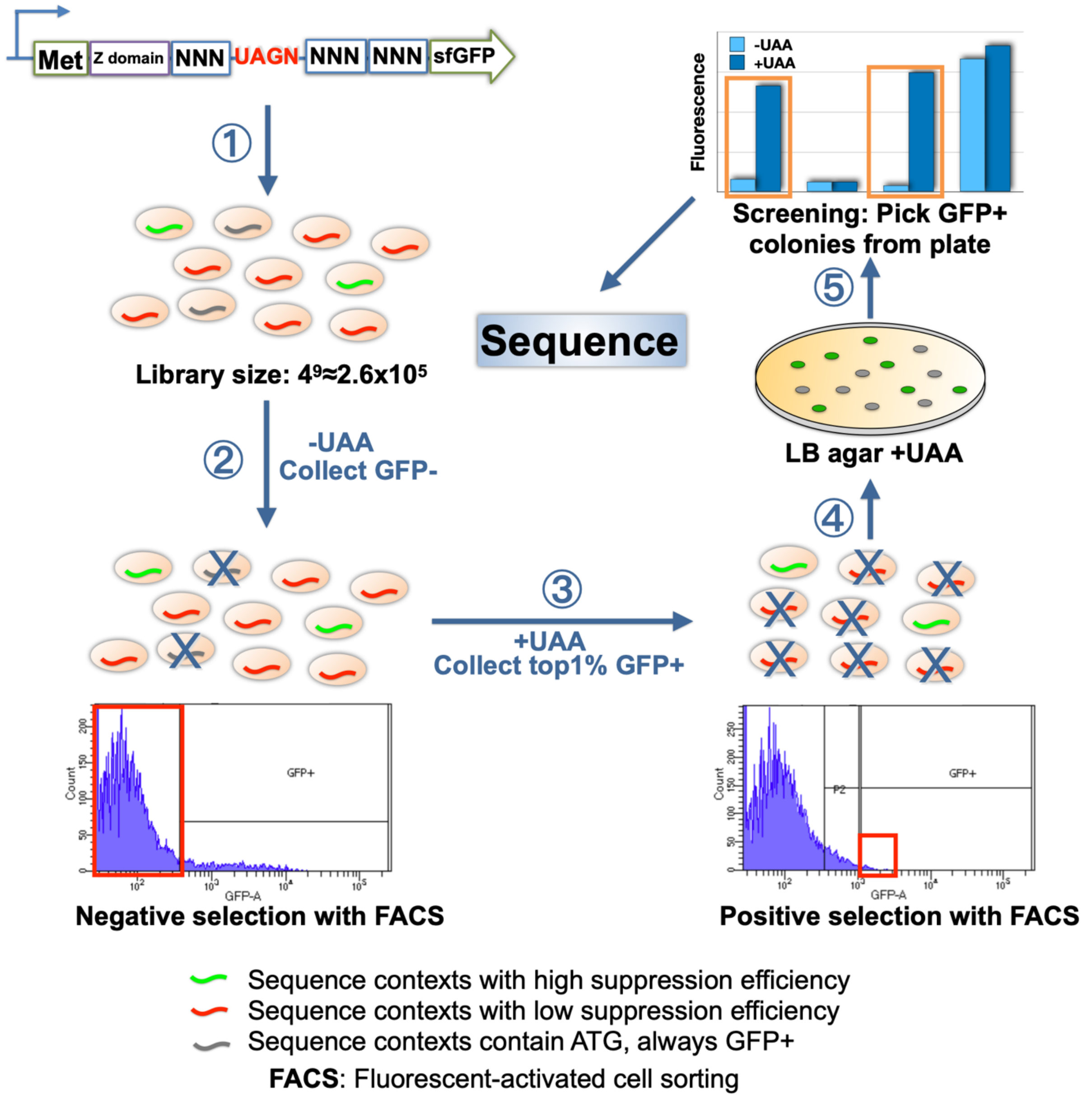

Supplementary Figure 2 Fluorescence-activated cell sorting (FACS)-based screening of recoding signals. Libraries (Supplementary Figure 1) were transformed into *E. coli* cells. The resulting cells were sorted based on sfGFP fluorescence (non-fluorescent cells were collected in the negative selection; cells with the top 1% of fluorescence intensities were collected in the positive selection). Populations inside red frame were collected in each step. The collected cells were plated and validated with fluorescence assays. Clones that were able to efficiently incorporate ncAA were sequenced.

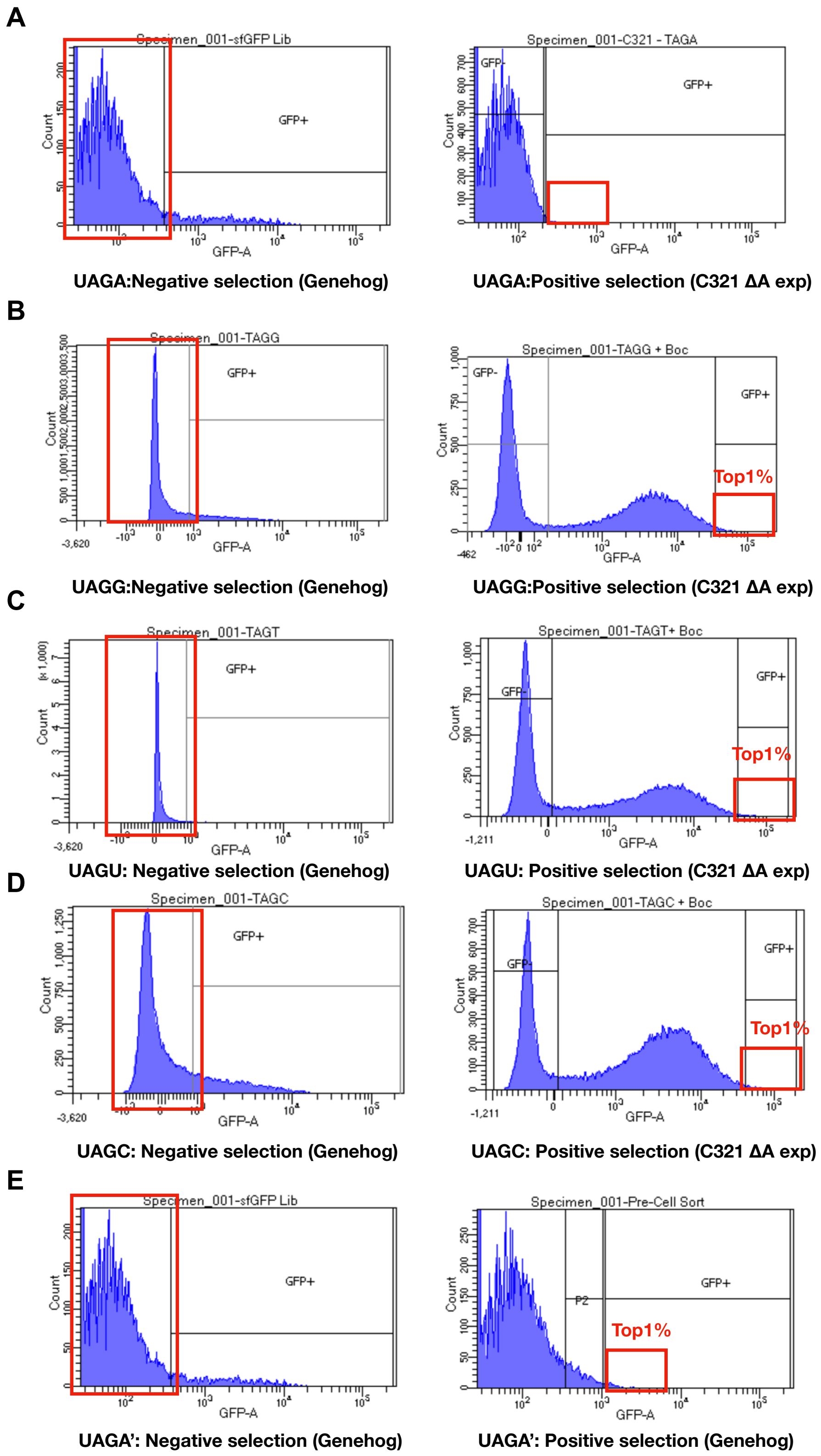
A
Specimen_001-sfGFP Lib
GFP+
Count
GFP-A
UAGA:Negative selection (Genehog)
Specimen_001-C321 - TAGA
GFP-
GFP+
UAGA:Positive selection (C321 ΔA exp)
B
Specimen_001-TAGG
UAGG:Negative selection (Genehog)
Specimen_001-TAGG + Boc
Top1%
UAGG:Positive selection (C321 ΔA exp)
C
Specimen_001-TAGT
UAGU: Negative selection (Genehog)
Specimen_001-TAGT+ Boc
UAGU: Positive selection (C321 ΔA exp)
D
Specimen_001-TAGC
UAGC: Negative selection (Genehog)
Specimen_001-TAGC + Boc
UAGC: Positive selection (C321 ΔA exp)
E
Specimen_001-sfGFP Lib
UAGA': Negative selection (Genehog)
Specimen_001-Pre-Cell Sort
P2
UAGA': Positive selection (Genehog)

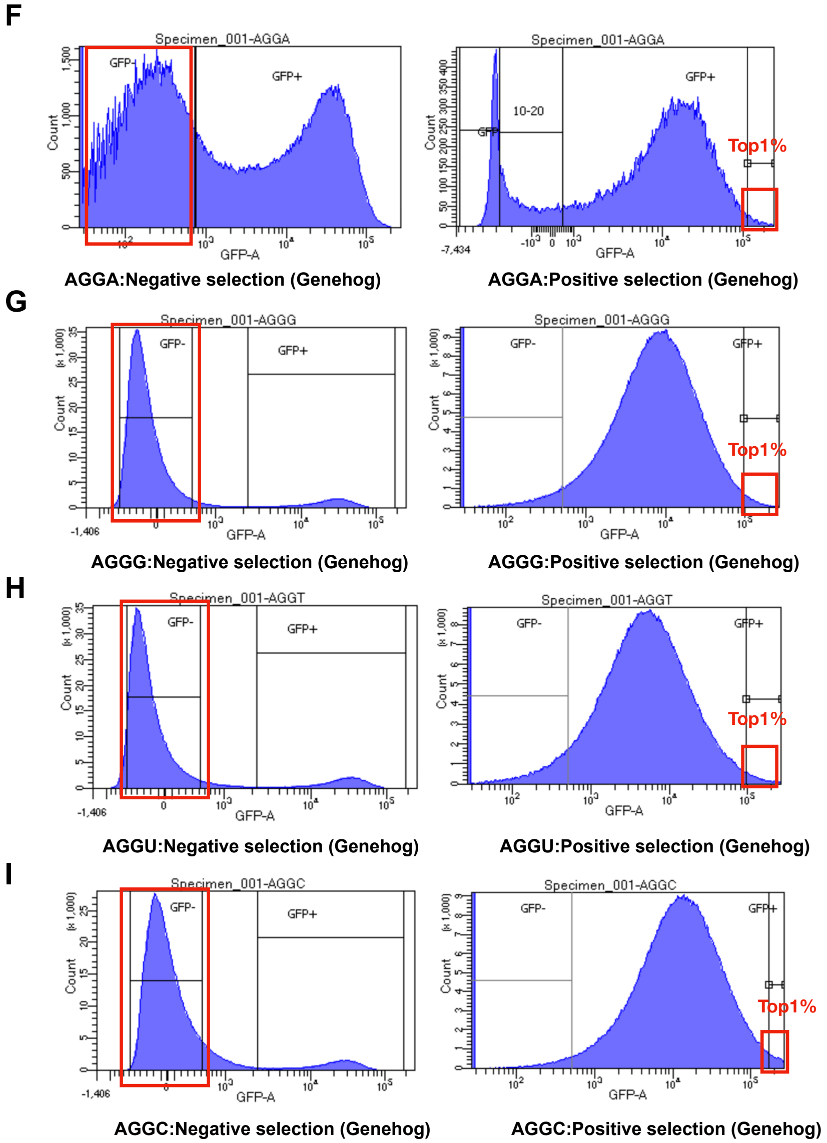

Supplementary Figure 3. (A)-(D) FACS analysis of Lib-UAGN-2. **(E)** FACS analysis of Lib-UAGA-1. (**F)-(I)** FACS analysis of Lib-AGGN. Populations labeled in the red boxes were collected (non-fluorescent cells were collected in the negative selection; cells with the top 1% of fluorescence intensities were collected in the positive selection). 10^6 events were recorded for each FACS analysis.

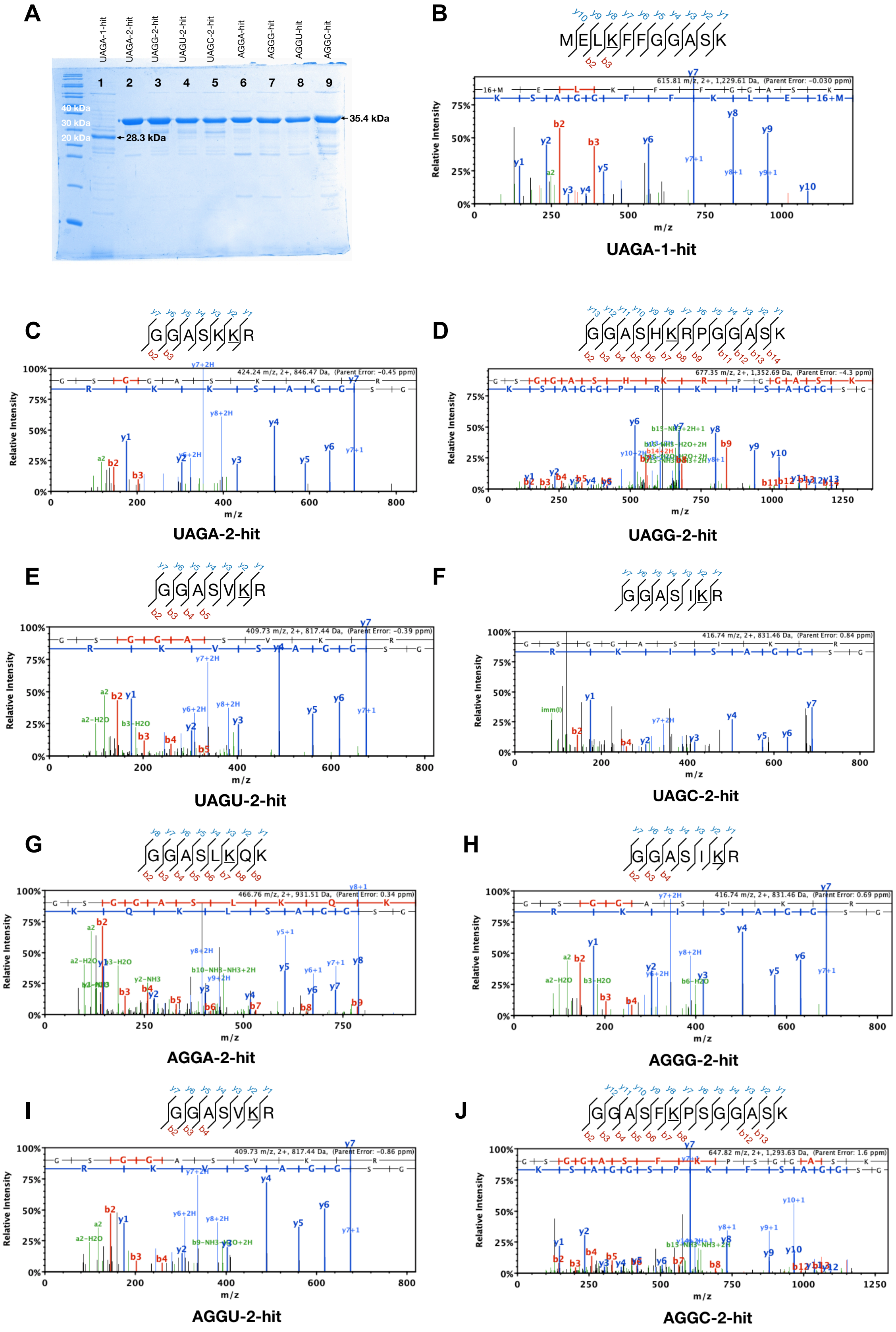
A
UAGA-1-hit
UAGA-2-hit
UAGG-2-hit
UAGU-2-hit
UAGC-2-hit
AGGA-hit
AGGG-hit
AGGU-hit
AGGC-hit
1 2 3 4 5 6 7 8 9
40 kDa
30 kDa
20 kDa
28.3 kDa
35.4 kDa
B
MELKFFGGASK
615.81 m/z, 2+, 1,229.61 Da, (Parent Error: -0.030 ppm)
Relative Intensity
m/z
UAGA-1-hit
C
GGASKKR
424.24 m/z, 2+, 846.47 Da, (Parent Error: -0.45 ppm)
UAGA-2-hit
D
GGASHKRPGGASK
677.35 m/z, 2+, 1,352.69 Da, (Parent Error: -4.3 ppm)
UAGG-2-hit
E
GGASVKR
409.73 m/z, 2+, 817.44 Da, (Parent Error: -0.39 ppm)
UAGU-2-hit
F
GGASIKR
416.74 m/z, 2+, 831.46 Da, (Parent Error: 0.84 ppm)
UAGC-2-hit
G
GGASLKQK
466.76 m/z, 2+, 931.51 Da, (Parent Error: 0.34 ppm)
AGGA-2-hit
H
GGASIKR
416.74 m/z, 2+, 831.46 Da, (Parent Error: 0.69 ppm)
AGGG-2-hit
I
GGASVKR
409.73 m/z, 2+, 817.44 Da, (Parent Error: -0.86 ppm)
AGGU-2-hit
J
GGASFKPSGGASK
647.82 m/z, 2+, 1,293.63 Da, (Parent Error: 1.6 ppm)
AGGC-2-hit

Supplementary Figure 4. Mass spectrometry analysis of BocLys incorporation in response to UAGN codons. (**A**) SDS-PAGE analysis of proteins purified from *E. coli* C321.ΔA.exp (for UAGA-1-hit and UAGN-2-hits) or *E. coli* GeneHogs (for AGGN-1-hits). The calculated molecular weight of sfGFP-UAGA-1-hit is 28.3 kDa. The calculated molecular weight of Z domain-sfGFP-UAGA-2-hits/AGGN-hits was 35.4 kDa. (**B**) Mass spectrometry analysis of sfGFP-UAGA-1-hit. (**C**)-(**F**) Mass spectrometry analysis of Z domain-sfGFP-UAGN-2-hits. (**G**) to (**J**) Mass spectrometry analysis of Z domain-sfGFP-AGGN-hits. Lysine (underlined in the peptide sequence), instead of BocLys, was observed at the target position. Since the BocLysRS cannot charge its tRNA with lysine according to both literature report (6) and our previous work (1), the observed peptide that contains lysine at the target site must be derived from the cleavage of the Boc group under the mass spectrometry conditions. The carbamate cleavage of BocLys was also observed previously with electron spray ionization process in the literature (7,8). The site-specific incorporation of ncAA in response to quadruplet codons is also consistent with our observation that significantly higher protein expression was observed in the presence of ncAA that in the absence of ncAA (Figure 4B).

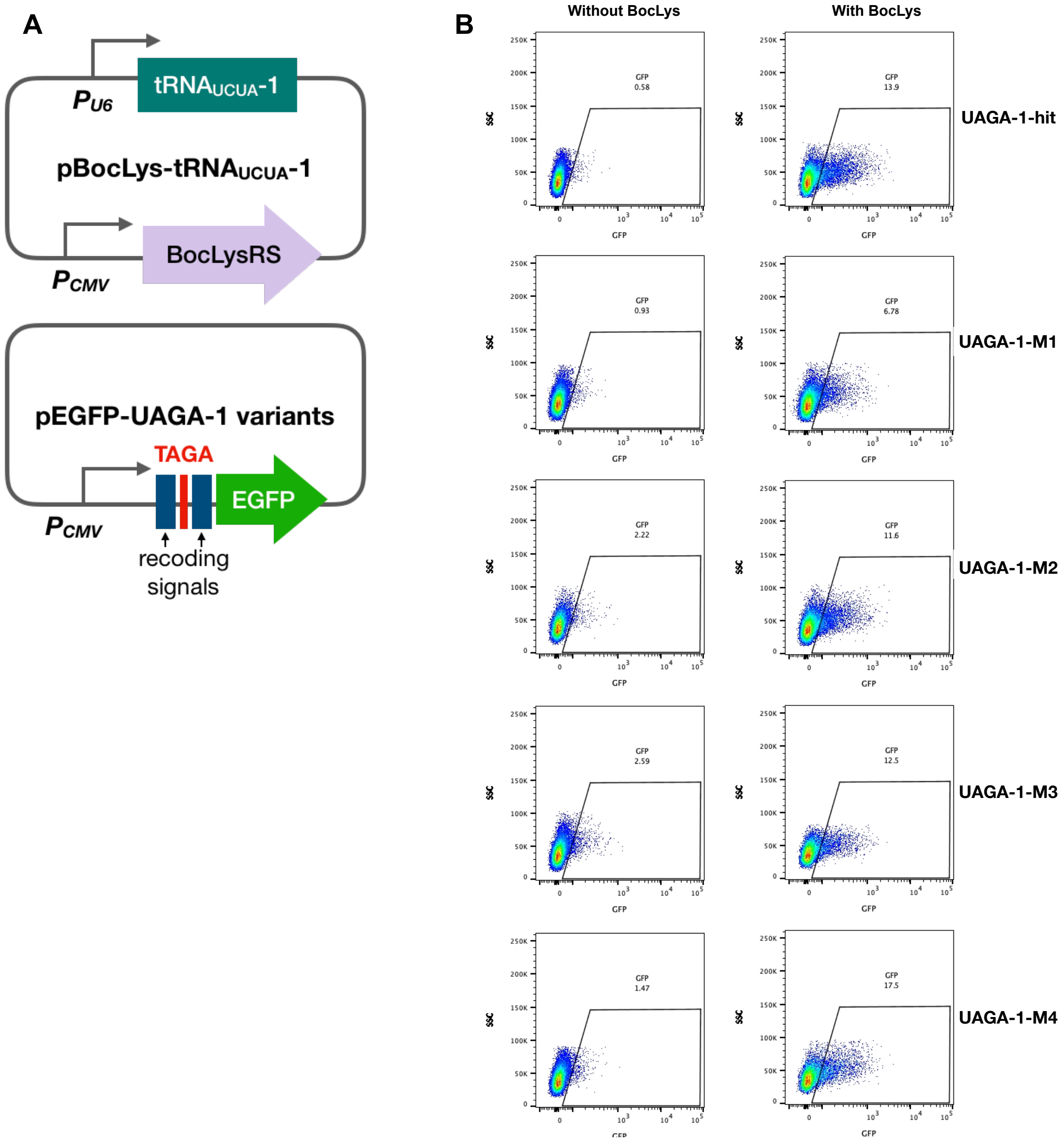

Supplementary Figure 5. Characterization of UAGA recoding signal in 297T cells. (**A**) Double-plasmid system for expressing UAGA codon flanking with UAGA-1-hit recoding signal and four mutants in EGFP-encoding gene in 293T cells (Figure 1G). (**B**) Density plots of flow cytometry analysis in Figure 1G. For each experiment, the same number (3×10^4) of 293T cells were sorted.

A

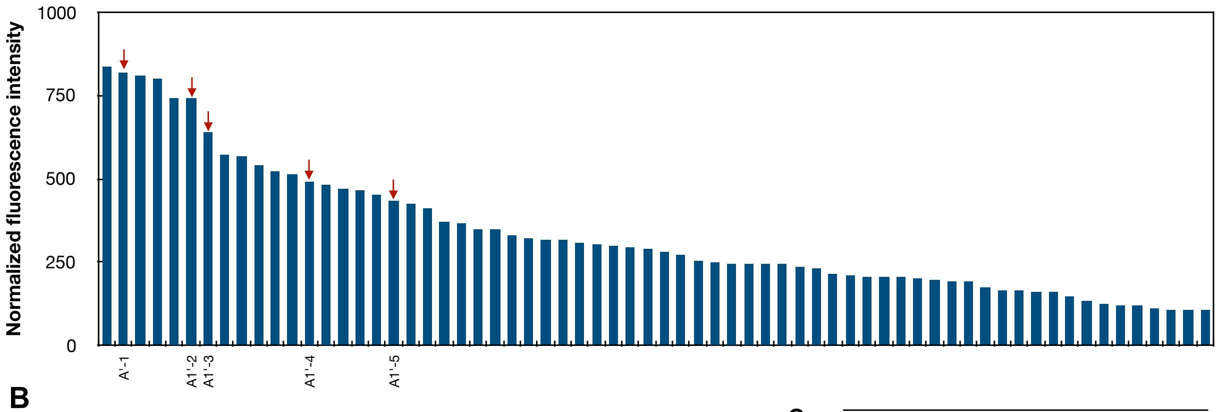

B

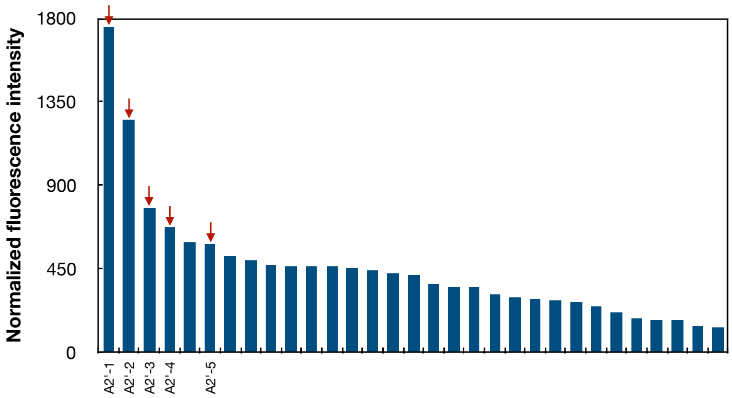

C

Name	Sequence		
	-3 to -1	UAGA	+5 to +7
A1'-1	**UUA**	UAGA	UGG
A1'-2	**UUA**	UAGA	UUG
A1'-3	**UUA**	UAGA	AUU
A1'-4	**UUA**	UAGA	UCA
A1'-5	**UUA**	UAGA	UUU
A2'-1	UUA	UAGA	**UUU**
A2'-2	UUA	UAGA	**UUU**
A2'-3	UGG	UAGA	**UUU**
A2'-4	CUA	UAGA	**UUU**
A2'-5	UUG	UAGA	**UUU**

Supplementary Figure 6. (**A**) Fluorescence assays of 66 positive clones identified from 96 randomly picked Lib-UAGA-1' colonies. (**B)** Fluorescence assays of 31 positive clones identified from 96 randomly picked Lib-UAGA-1'' colonies. Normalized fluorescence intensity is calculated by fluorescence intensity/OD_{600}. (**C**) Recoding signal from each library respectively. Converged nucleotides are labeled in bold. Sequenced clones in (C) were highlighted with red arrows in (A) and (B).

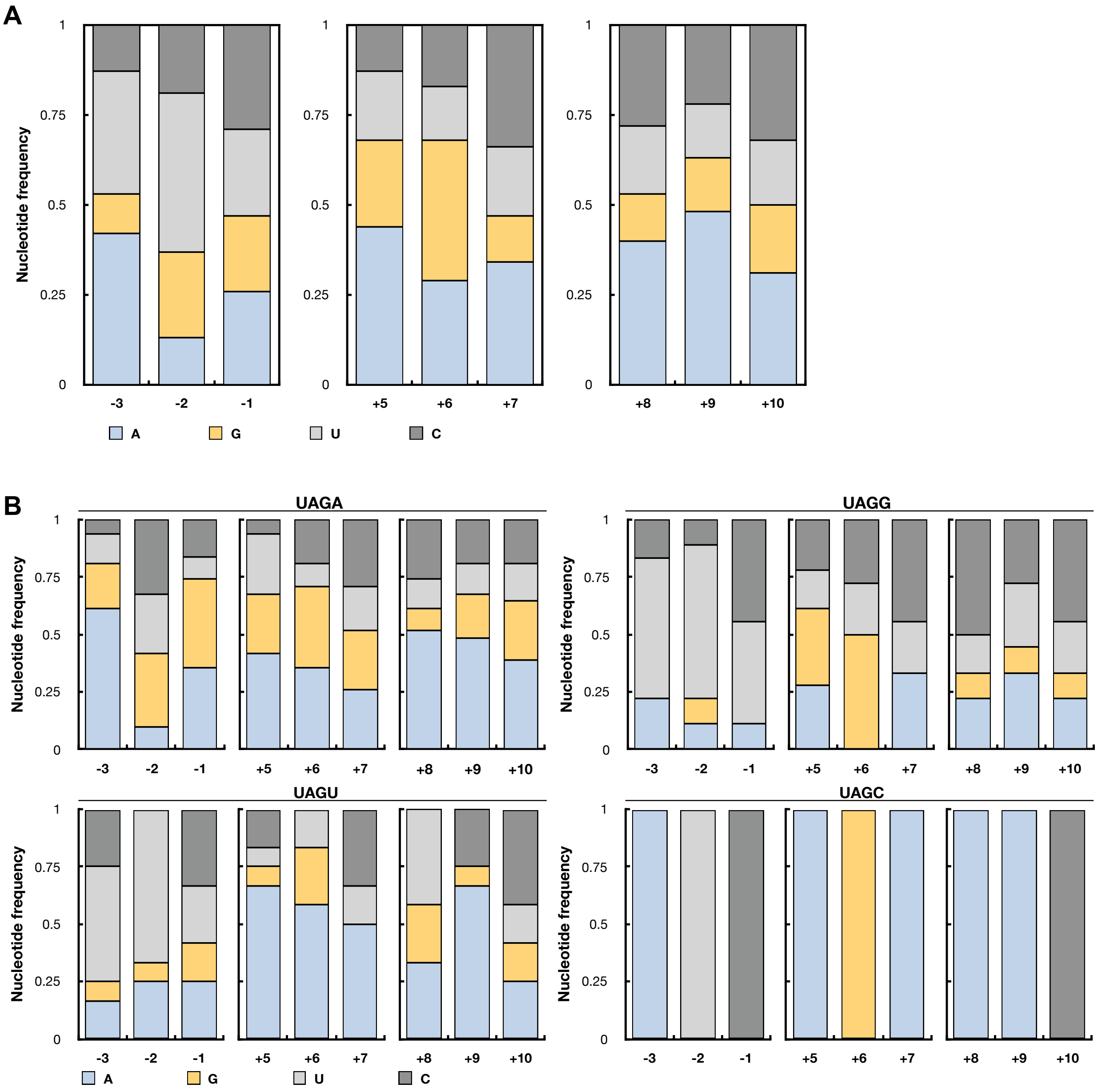

Supplementary Figure 7 (**A**) Nucleotide sequence analysis of 62 unique hits (Supplementary Table 3) identified from Lib-UAGN-2. *(***B**) Nucleotide sequence analysis of unique recoding signals for each UAGN decoding (Supplementary Table 4) identified from Lib-UAGN-2.

A

Name	Sequence			
	-3 to -1	UAGN	+5 to +7	+8 to +10
UAGA-M5	UUU	UAGA	AGA	AUC
UAGG-M5	UUU	UAGG	AGA	CCU
UAGU-M5	UUU	UAGU	AGA	ACU
UAGC-M5	UUU	UAGC	AGA	AAC

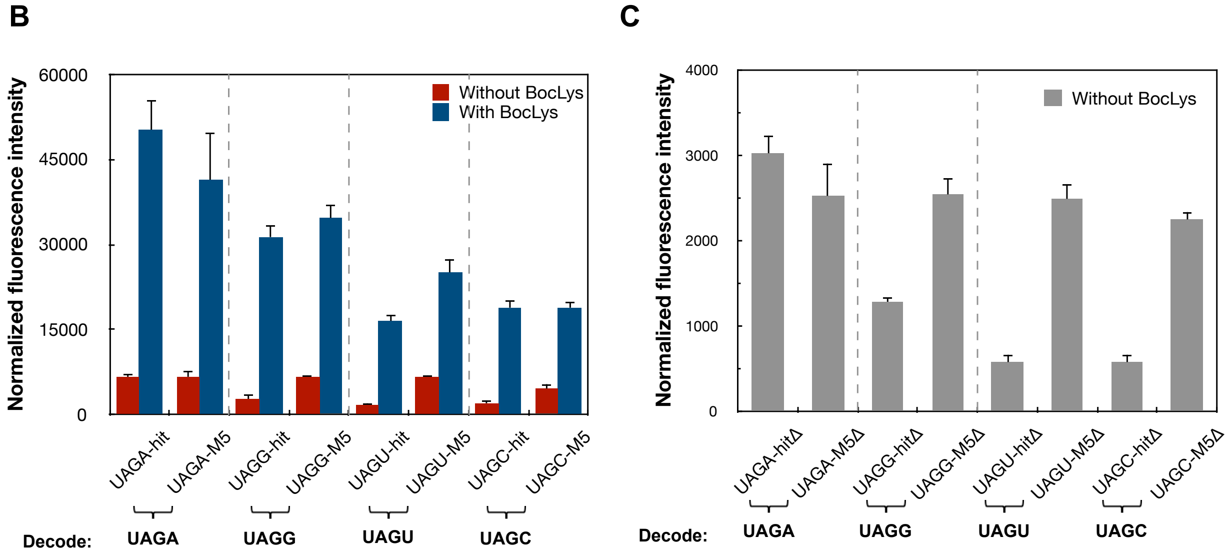

Supplementary Figure 8. (A) Sequence of UAGN-2-M5. In UAGN-2-M5, the -3 to -1 region was mutated to UUU with downstream sequence the same as UAGN-2-hits. Mutations in each variant are labeled in red. (**B**) Fluorescence assays of UAGAn-2-hit and UAGN-2-M5. (**C**) Fluorescence assays of UAGN-2-hitΔ and UAGN-2-M5Δ mutants (tRNA$_{UCUA}$-1 and BocLysRS were removed from UAGN-2-hit and UAGN-2-M5 constructs, respectively). The expressions were conducted either in the absence or presence of 5mM BocLys. Normalized fluorescence intensity is calculated by fluorescence intensity/OD$_{600}$. Each data point is the average of triplicate measurement with standard deviation. Raw OD$_{600}$ values were shown in Supplementary Figure 10E.

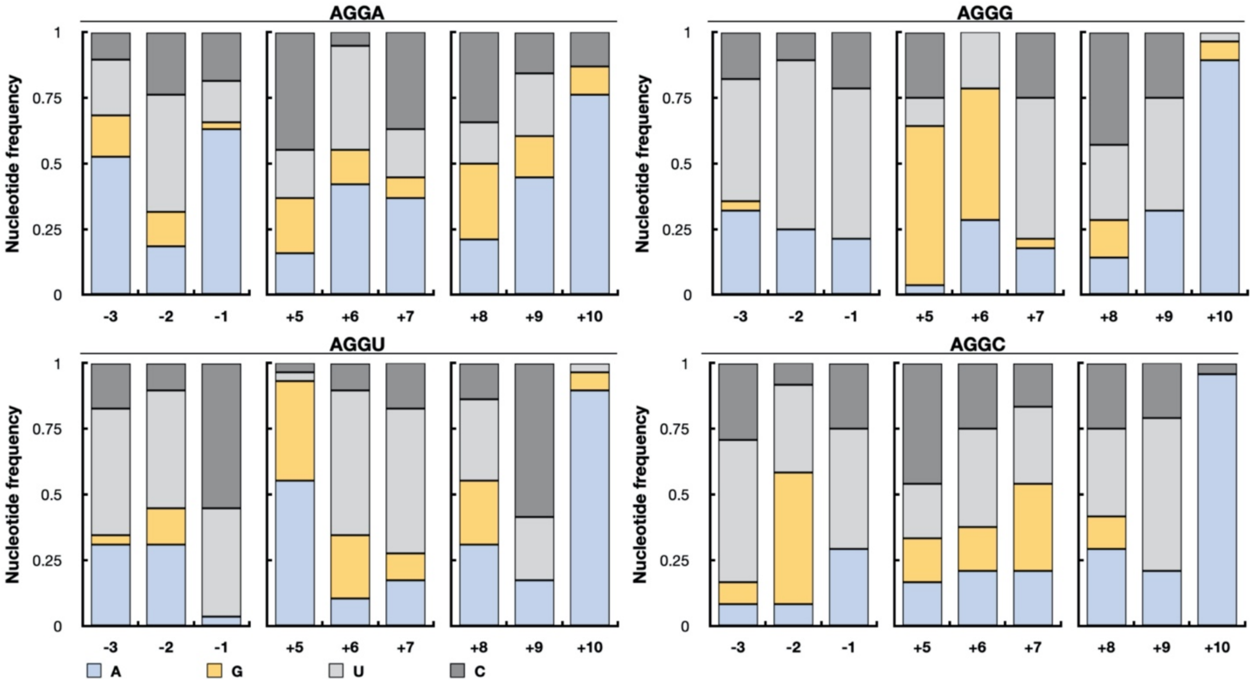

Supplementary Figure 9. Nucleotide sequence analysis of recoding signals for AGGN decoding.

A

Name	OD$_{600}$		STDEV (-BocLys)	STDEV (+BocLys)
	-BocLys	+BocLys		
sfGFP-wt'	0.665	/	0.006	/
UAGA-1-hit	0.826	0.821	0.003	0.005
UAGA-1-M1	0.716	0.706	0.005	0.006
UAGA-1-M2	0.687	0.691	0.005	0.006
UAGA-1-M3	0.907	0.899	0.004	0.008
UAGA-1-M4	0.578	0.569	0.005	0.005

B

Name	OD$_{600}$		STDEV (-BocLys)	STDEV (+BocLys)
	-BocLys	+BocLys		
sfGFP-wt	0.513	/	0.071	
UAGA-2-hit	0.398	0.446	0.027	0.071
UAGA-2-M1	0.407	0.422	0.062	0.067
UAGG-2-hit	0.444	0.349	0.077	0.051
UAGG-2-M1	0.418	0.380	0.055	0.050
UAGU-2-hit	0.353	0.418	0.060	0.037
UAGU-2-M1	0.377	0.418	0.066	0.037
UAGC-2-hit	0.433	0.391	0.083	0.019
UAGC-2-M1	0.377	0.415	0.045	0.031

C

Name	OD$_{600}$		STDEV (-BocLys)	STDEV (+BocLys)
	-BocLys	+BocLys		
UAGA-2-M2	0.449	0.441	0.016	0.059
UAGA-2-M3	0.407	0.486	0.005	0.027
UAGA-2-M4	0.408	0.462	0.015	0.013
UAGG-2-M2	0.419	0.380	0.021	0.027
UAGG-2-M3	0.423	0.393	0.015	0.027
UAGG-2-M4	0.424	0.406	0.058	0.018
UAGU-2-M2	0.369	0.415	0.054	0.005
UAGU-2-M3	0.371	0.354	0.076	0.027
UAGU-2-M4	0.393	0.409	0.060	0.032
UAGC-2-M2	0.390	0.395	0.062	0.067
UAGC-2-M3	0.385	0.375	0.037	0.032
UAGC-2-M4	0.387	0.394	0.044	0.021

D

Name	OD$_{600}$					STDEV (-BocLys)	STDEV (+BocLys)	STDEV (-ncAA)	STDEV (+K-alkyne)	STDEV (+K-Alkene)
	-BocLys	+BocLys	-ncAA	+K-alkyne	+K-alkene					
UAGA-1-hit	0.347	0.340	0.387	0.381	0.478	0.026	0.022	0.039	0.039	0.005
UAGA-2-hit	0.478	0.557	0.653	0.771	0.802	0.039	0.058	0.029	0.014	0.001
UAGG-2-hit	0.518	0.641	0.614	0.765	0.786	0.018	0.001	0.005	0.051	0.009
UAGU-2-hit	0.565	0.688	0.604	0.752	0.722	0.004	0.026	0.017	0.040	0.008
UAGC-2-hit	0.570	0.664	0.595	0.720	0.705	0.020	0.021	0.004	0.028	0.017

E

Name	OD$_{600}$		STDEV (-BocLys)	STDEV (+BocLys)
	-BocLys	+BocLys		
UAGA-1-hit	0.295	0.387	0.013	0.006
UAGA-2-hit	0.327	0.340	0.037	0.002
UAGA-wt	0.310	0.367	0.008	0.008
UAGG-2-hit	0.308	0.366	0.020	0.003
UAGG-wt	0.309	0.348	0.019	0.021
UAGU-2-hit	0.315	0.350	0.027	0.005
UAGU-wt	0.282	0.338	0.013	0.030
UAGC-2-hit	0.307	0.362	0.044	0.013
UAGC-wt	0.273	0.338	0.002	0.010

F

Name	OD$_{600}$		STDEV (-BocLys)	STDEV (+BocLys)
	-BocLys	+BocLys		
AGGA-hit	0.714	0.699	0.083	0.063
AGGA-M1	0.819	0.808	0.027	0.063
AGGG-hit	0.758	0.679	0.019	0.059
AGGG-M1	0.836	0.716	0.010	0.013
AGGU-hit	0.748	0.684	0.038	0.052
AGGU-M1	0.827	0.734	0.045	0.020
AGGC-hit	0.788	0.766	0.051	0.091
AGGC-M1	0.646	0.694	0.018	0.036

G

Name	OD$_{600}$		STDEV (-BocLys)	STDEV (+BocLys)
	-BocLys	+BocLys		
UAGA-M5	0.467	0.525	0.006	0.047
UAGA-hitΔ	0.677	/	0.024	/
UAGA-M5-Δ	0.725	/	0.064	/
UAGG-M5	0.444	0.403	0.016	0.025
UAGG-hitΔ	0.665	/	0.020	/
UAGG-M5-Δ	0.737	/	0.018	/
UAGU-M5	0.385	0.400	0.019	0.005
UAGU-hitΔ	0.651	/	0.062	/
UAGU-M5-Δ	0.747	/	0.024	/
UAGC-M5	0.463	0.441	0.044	0.031
UAGC-hitΔ	0.673	/	0.013	/
UAGC-M5-Δ	0.738	/	0.018	/

Supplementary Figure 10. Raw OD$_{600}$ values **(A)** for Figure 1E; **(B)** for Figure 3A; (**C**) for Figure 3B; **(D)** for Figure 4B; **(E)** for Figure 4C; (**F**) for Figure 5E; (**G**) for Supplementary Figure 8A and Figure 8B. Each data is the average of triplicate measurement.

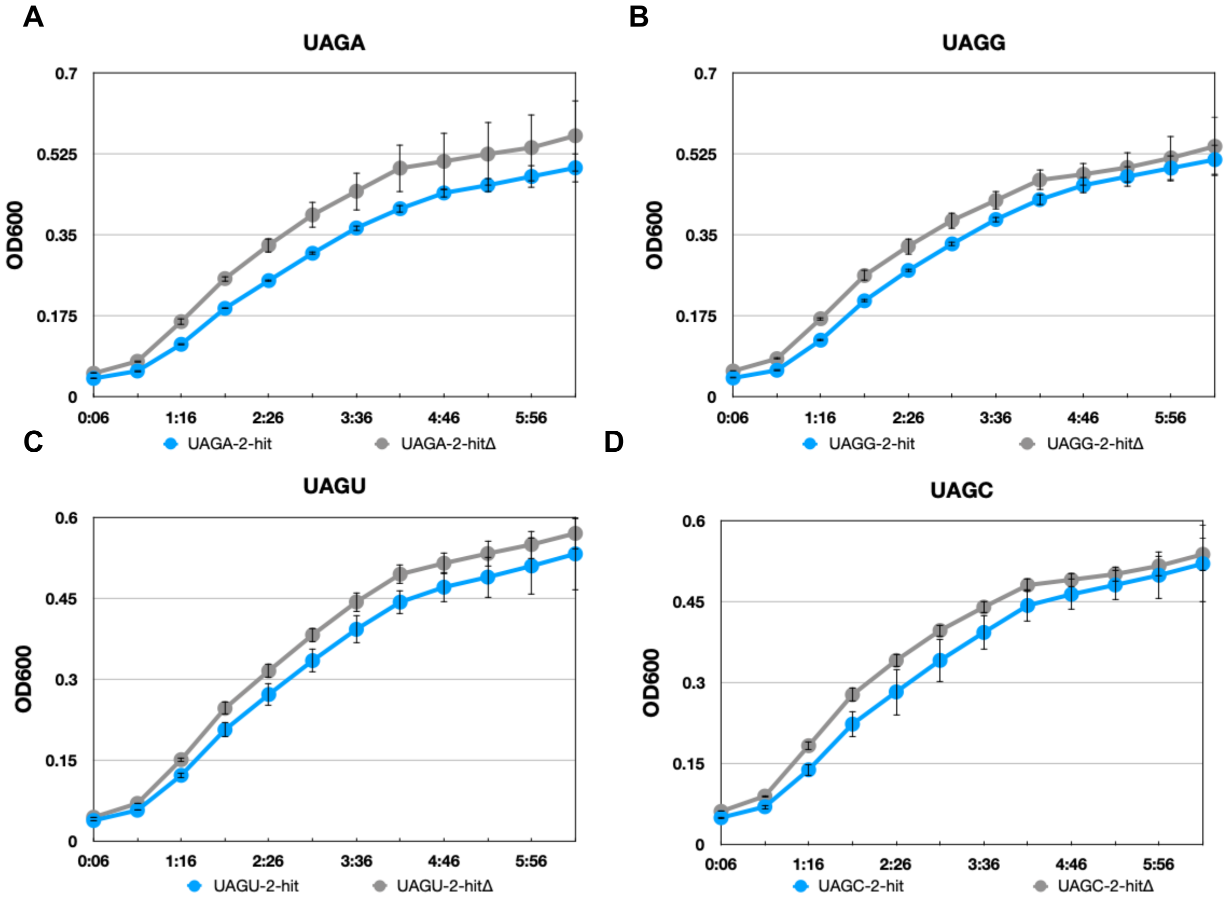

Supplementary Figure 11. (**A**)-(**D**) Apparent growth defect was not detected with the UAGN-decoding system in *E. coli* GeneHogs host. The growth rate of UAGN-2-hit variants were similar to UAGN-2-hitΔ variants ($tRNA_{UCUA}$-1 and BocLysRS were removed from UAGN-2-hit). BocLys (5 mM) was provided in the media. The X-axis shows the time of cultivation (h). Each data point is the average of triplicate measurement with standard deviation.

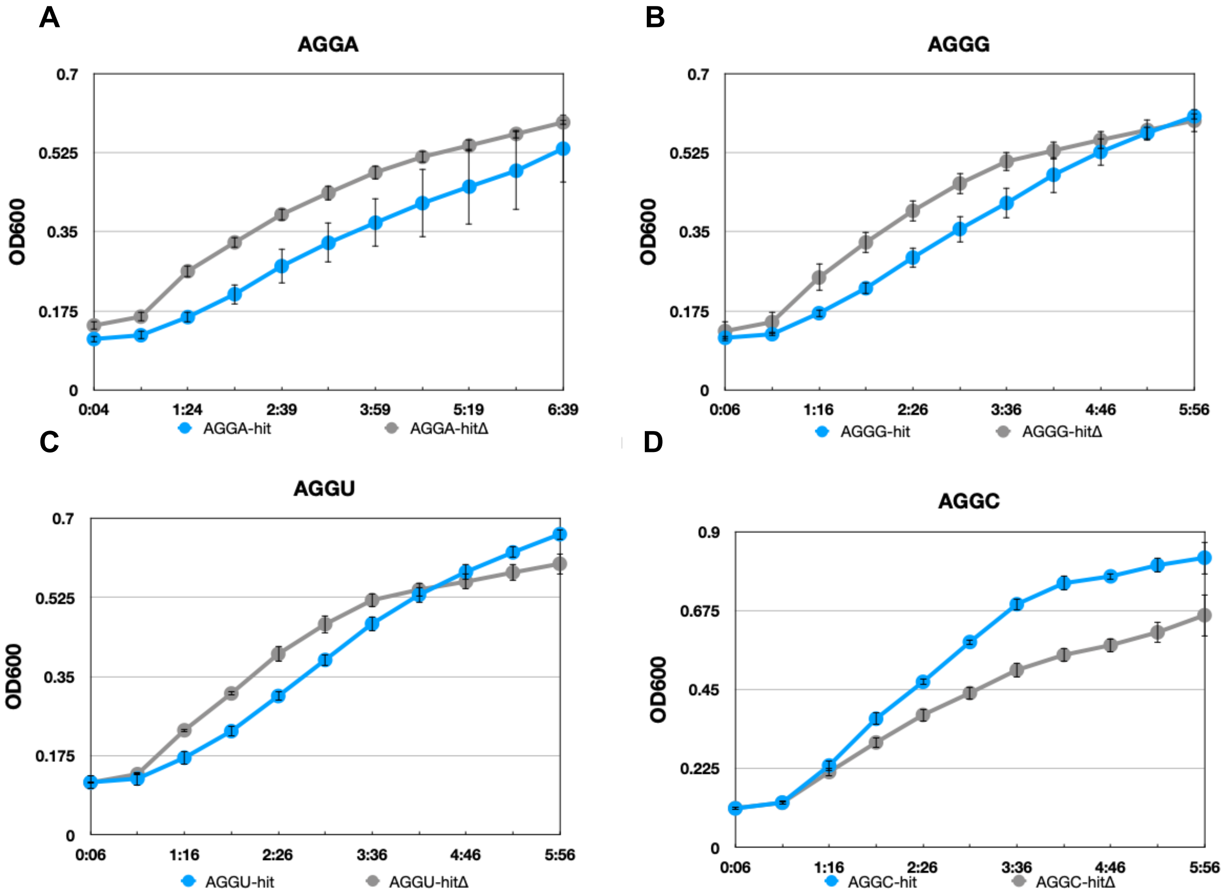

Supplementary Figure 12. (**A**)-(**D**) Apparent growth defect was not detected with the AGGN-decoding in *E. coli* GeneHogs host. The growth rate of UAGN-2-hit variants were similar to AGGN-2-hitΔ variants ($tRNA_{UCCU}$-M7 and BocLysRS were removed from AGGN-2-hit). BocLys (5 mM) was provided in the media. The X-axis shows the time of cultivation (h). Each data point is the average of triplicate measurement with standard deviation.

Reference

1. Wang, N., Shang, X., Cerny, R., Niu, W. and Guo, J. (2016) Systematic evolution and study of UAGN decoding tRNAs in a genomically recoded bacteria. *Sci. Rep.*, **6**, 21898.
2. Chen, Y., Wan, Y., Wang, N., Yuan, Z., Niu, W., Li, Q. and Guo, J. (2018) Controlling the replication of a genomically recoded HIV-1 with a functional quadruplet codon in mammalian cells. *ACS Synth. Biol.*, **7**, 1612-1617.
3. Tikole, S. and Sankararamakrishnan, R. (2006) A survey of mRNA sequences with a non-AUG start codon in RefSeq database. *J. Biomol. Struct. Dyn.*, **24**, 33-41.
4. Guo, J., Melançon III, C.E., Lee, H.S., Groff, D. and Schultz, P.G. (2009) Evolution of Amber Suppressor tRNAs for Efficient Bacterial Production of Proteins Containing Nonnatural Amino Acids. *Angewandte Chemie International Edition*, **48**, 9148-9151.
5. Niu, W., Schultz, P.G. and Guo, J. (2013) An expanded genetic code in mammalian cells with a functional quadruplet codon. *ACS Chem. Biol.*, **8**, 1640-1645.
6. Yanagisawa, T., Ishii, R., Fukunaga, R., Kobayashi, T., Sakamoto, K. and Yokoyama, S. (2008) Multistep Engineering of Pyrrolysyl-tRNA Synthetase to Genetically Encode N ϵ-(o-Azidobenzyloxycarbonyl) lysine for Site-Specific Protein Modification. *Chem. Biol. (Cambridge, MA, U. S.)*, **15**, 1187-1197.
7. Nguyen, D.P., Mahesh, M., Elsasser, S.J., Hancock, S.M., Uttamapinant, C. and Chin, J.W. (2014) Genetic Encoding of Photocaged Cysteine Allows Photoactivation of TEV Protease in Live Mammalian Cells. *J. Am. Chem. Soc.*, **136**, 2240-2243.
8. Schmied, W.H., Elsasser, S.J., Uttamapinant, C. and Chin, J.W. (2014) Efficient multisite unnatural amino acid incorporation in mammalian cells via optimized Pyrrolysyl tRNA Synthetase/tRNA expression and engineered eRF1. *J. Am. Chem. Soc.*, **136**, 15577-15583.
